# Structural, genetic, and adaptive basis of superhydrophobicity in rice leaves

**DOI:** 10.1093/plphys/kiag514

**Published:** 2026-07-23

**Authors:** Tian Zhu, Asuka Hiraiwa, Saori Aiga, Manaki Mimura, Takanori Yoshikawa, Yutaka Sato, Jun-ichi Itoh

**Affiliations:** Graduate School of Agricultural and Life Sciences, The University of Tokyo, Tokyo 113-8657, Japan; Graduate School of Agricultural and Life Sciences, The University of Tokyo, Tokyo 113-8657, Japan; Graduate School of Agricultural and Life Sciences, The University of Tokyo, Tokyo 113-8657, Japan; Graduate School of Agricultural and Life Sciences, The University of Tokyo, Tokyo 113-8657, Japan; National Institute of Genetics, Mishima, Shizuoka 411-8540, Japan; National Institute of Genetics, Mishima, Shizuoka 411-8540, Japan; Graduate School of Agricultural and Life Sciences, The University of Tokyo, Tokyo 113-8657, Japan

## Abstract

In most plants, leaf surfaces exhibit water-repellent properties, which protect against pathogens and weather. Plants such as lotus and rice have evolved outstanding water-repellent properties, termed superhydrophobicity (static contact angle >150°). However, the structural and genetic basis of these properties in crop plants remains unclear. In this study, water repellency was quantified by measuring static contact angles, and its relationship with epicuticular wax load, the extent of wax coverage assessed by scanning electron microscopy, and the differentiation of papillae was analyzed in a large collection of rice wetting-leaf mutants and diverse cultivars, as well as wild *Oryza* species. Analysis of leaf surface structure in mutants derived from cultivated rice revealed that the degree of water repellency largely depends on the cuticular wax quantity and quality. Although papillae differentiation contributes minorly to water repellency, it is suggested to play a crucial role in achieving superhydrophobicity. These morphological characteristics are regulated by at least 5 previously characterized genes. Furthermore, we found that superhydrophobicity is widely conserved among cultivated rice and is also present in ancestral species. These observations suggest that superhydrophobicity evolved independently of rice domestication. These findings provide a structural and genetic framework for understanding the evolution and potential adaptive significance of leaf superhydrophobicity in rice and offer insights for engineering leaf-surface traits relevant to crop resilience and management.

## Introduction

In plants, the surface cuticle is responsible for plant–environment interactions and plays an important role in defense against environmental and biological stresses such as drought, ultraviolet radiation, pathogens, and insects (Koch and Ensikat 2008; Yeats and Rose 2013; Barthlott et al. 2017; Jolliffe et al. 2023). One of the physical properties associated with the cuticle is water repellency. The degree of water repellency varies among plant species, but most plants exhibit very good water-repellent properties (Barthlott et al. 2017). Water repellency facilitates gas exchange through stomata by maintaining contact with the atmosphere when rainwater adheres to leaf surfaces. It also hinders the attachment of pathogens to the epidermis, thereby conferring protection against disease (Barthlott et al. 2017).

Epicuticular wax deposited on the leaf surface is an important contributor to water repellency (Koch and Ensikat 2008; Yeats and Rose 2013; Barthlott et al. 2017; Jolliffe et al. 2023). This epicuticular wax consists of compounds derived from very-long-chain fatty acids (VLCFAs; C20–C34), including alkanes, aldehydes, primary and secondary alcohols, ketones, and esters, although the precise composition varies for different plant species, organs, growth stages, and conditions (Yeats and Rose 2013; Lee and Suh 2015; Batsale et al. 2021). VLCFAs are synthesized in the endoplasmic reticulum by fatty acid elongation (FAE) complex enzymes, including β-ketoacyl-CoA synthase (KCS), β-ketoacyl-CoA reductase (KCR), β-hydroxy acyl-CoA dehydratase, and enoyl-CoA reductase (Kunst and Samuels 2009; Lee and Suh 2015). The elongated VLCFA-CoA is processed by an alkane-forming or alcohol-forming pathway to generate a cuticular wax mixture, which is eventually secreted (Kunst and Samuels 2009; Yeats and Rose 2013; Lee and Suh 2015). The secreted cuticular wax forms epicuticular crystals that are deposited on the surface of plants (Koch and Ensikat 2008). These wax crystals may be crucial for leaf-surface water repellency (Bhushan 2012; Barthlott et al. 2017).

Some plants exhibit outstanding water-repellent properties associated with additional functions, so-called superhydrophobicity. Lotus (*Nelumbo nucifera*) is one such species (Guo and Liu 2007; Bhushan 2012; Barthlott et al. 2017). Superhydrophobic leaf surfaces have micro-protrusions and wax crystals (Barthlott and Neinhuis 1997; Barthlott et al. 2017) that create static contact angles on the leaf surface greater than 150°. In a plant species with a superhydrophobic surfacecombined with low hysteresis, droplets of water roll across the surface and wash away dirt. This self-cleaning property maintains gas exchange through stomata, increases photosynthetic efficiency, and is called “the lotus effect” (Barthlott and Neinhuis 1997; Neinhuis and Barthlott 1997; Bhushan 2012; Barthlott et al. 2017; Yeats and Rose 2013). Rice plants also exhibit superhydrophobicity, with wax crystals on the leaves and differentiated papillae, which are micro-protrusions on the epidermis (Neinhuis and Barthlott 1997; Guo and Liu 2007; Barthlott et al. 2017). Rice is often grown in paddy fields, and water repellency is important because it generates a gas film across the submerged epidermis (Raven 2008; Kurokawa et al. 2018).

The genes necessary for water repellency may be identified by analyzing mutants that are deficient in this property. One requirement for water repellency may be the synthesis of cuticular wax. Mutants deficient in the accumulation of such wax have been reported in various plant species (Yeats and Rose 2013; Lee and Suh 2015). In rice, several mutants with abnormal accumulation of cuticular wax exhibit reduced tolerance to environmental stress, as well as reduced water repellency (Yu et al. 2008; Islam et al. 2009; Qin et al. 2011; Mao et al. 2012; Gan et al. 2016, 2017; Zhang et al. 2016; Wang et al. 2017; Kurokawa et al. 2018). Among these, *wax crystal-sparse leaf* (*wsl*) mutants show reduced accumulation of epicuticular wax and abnormal cuticular wax composition in leaf. *WAX CRYSTAL-SPARSE LEAF1* (*WSL1*) encodes a KCS that catalyzes the biosynthesis of C24 from C20 VLCFAs (Yu et al. 2008). Another KCS, encoded by *WSL4*, is involved in the elongation of long (>C22) VLCFAs (Gan et al. 2017; Wang et al. 2017). *WSL3* encodes a KCR for a FAE complex that is essential for the elongation of VLCFAs and accumulation of leaf wax (Gan et al. 2016). *OsGL1–1/WSL2* is a homolog of the *Arabidopsis WAX2* and maize *GL1* gene, which does not encode a component of an FAE complex. It affects epicuticular wax production by participating in the elongation of VLCFAs, and the mutants show reduced water repellency (Qin et al. 2011; Mao et al. 2012). *OsHSD1*, a member of the hydroxysteroid dehydrogenases gene family in rice, is also involved in leaf epicuticular wax production and lipid homeostasis (Zhang et al. 2016). *Leaf Gas Film 1* (*LGF1*), the same gene as *OsHSD1*, is necessary for facilitating plant gas exchange under water (Kurokawa et al. 2018). The *dripping-wet leaf 7* (*drp7*) mutant of *LGF1* cannot maintain a gas film and exhibits reduced water repellency. *LGF1* regulates C30 primary alcohol synthesis, which is necessary to produce an abundance of epicuticular wax on leaf surfaces (Kurokawa et al. 2018).

Another factor that may be important for water repellency is the microstructure of leaf epidermal cells. Little is known about the underlying genetic mechanisms that create the microstructure of epidermal cells, but *BRIGHT GREEN LEAF* (*BGL*) is involved in the formation of papillae on rice leaves. *BGL* encodes OsRopGEF10, a member of the plant-specific RopGEF family of proteins, but its influence on water repellency has not been examined (Yoo et al. 2011).

Water repellency arises from physicochemical interactions between water and hierarchical epidermal structures and therefore reflects both surface chemistry, including wax composition and crystallization, and microtopography, such as papillae and cell shape (Koch and Ensikat 2008; Bhushan 2012; Barthlott et al. 2017). Although superhydrophobicity has been extensively discussed in the fields of biomimetics and surface physics, experimental genetic dissection within a single plant species remains limited. Rice provides a unique experimental system because it combines papillae with abundant wax crystals and is frequently cultivated under waterlogged conditions, in which leaf surface traits strongly influence gas-film formation and disease susceptibility (Kurokawa et al. 2018). In this study, we integrated contact-angle phenotyping, scanning electron microscopy (SEM)-based structural analysis, and genetic identification of causal loci in a large collection of rice wetting-leaf mutants, together with surveys of cultivated and wild *Oryza* species. This integrated approach identifies key structural determinants of leaf surface properties and 5 genes controlling them, thereby providing a practical framework for manipulating leaf surface traits to improve crop resilience and management.

## Results

### Relationship between surface structure and water repellency

**Figure 1:**
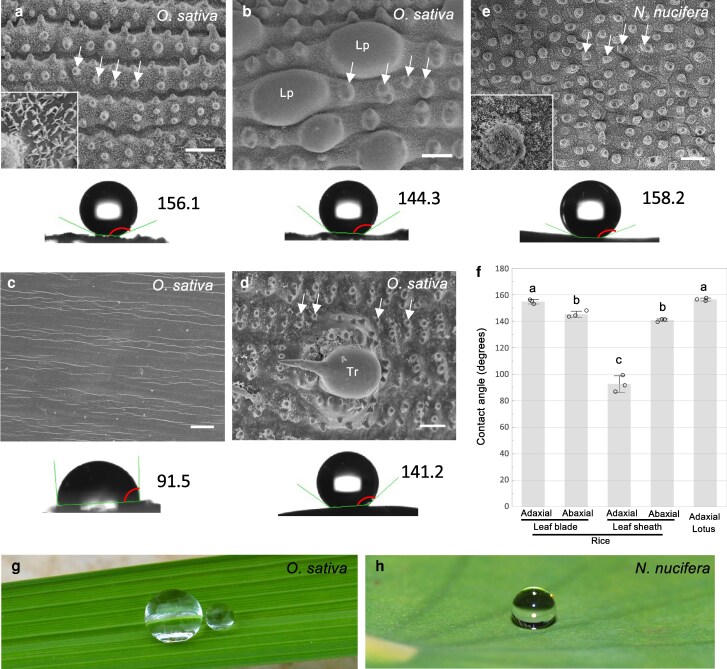
Surface structure and water repellency for different parts of the leaf. a) Scanning electron microscopy image of the adaxial surface of the 6th leaf blade of a rice (*Oryza sativa*) plant. b**)** Abaxial surface of the 6th leaf blade. c) Adaxial surface of the 6th leaf sheath. d**)** Abaxial surface of the 6th leaf sheath. e) Adaxial surface of the lotus (*Nelumbo nucifera*) leaf. Inset in (a) and (e) is an enlarged view of the surface. For each panel, the picture of the water droplet on the leaf surface and the measurement of the contact angle of the representative 1 are indicated. The red arc indicates the contact angle. White arrows in (a), (b), (d), and (e) indicate small papillae. Lp, large papilla; Tr, trichome. Scale bars in (a–d) = 10 µm, (e) = 20 µm. f) Contact angles of the leaf parts in rice and lotus. Bars represent mean ± SD (*n* = 3). Different letters above bars indicate statistically significant differences among the leaf parts based on Tukey's HSD test (*P* < 0.05). g) A rice leaf with water droplets on the adaxial surface. h) A lotus leaf with water droplets on the adaxial surface.

To understand the relationship between epidermal phenotypes (papillae and wax crystals) and water repellency, we studied 4 different parts of the rice (*O*. *sativa*) leaf using SEM. In rice, the adaxial and abaxial surfaces of the leaf blade (the strap-shaped upper portion of the leaf) correspond to the upper and lower lamina, respectively. By contrast, in the leaf sheath (the tubular portion enclosing the culm), the adaxial surface faces inward toward the culm, while the abaxial surface faces outward to the air. On the adaxial surface of the leaf blade, there were many uniformly distributed 1 to 2 µm papillary protrusions on the epidermal cell layer (Fig. 1a). The leaf surface was also covered with wax crystals (Fig. 1a, inset). On the abaxial surface of the leaf blade, in addition to papillae that were similar in size to those on the adaxial surface, papillae larger than 20 µm were also observed (Fig. 1b). These large papillae have been shown to be genetically distinct from the small papillae (Yoo et al. 2011); therefore, all papillae discussed in this study refer exclusively to the small papillae measuring 1 to 2 µm in size. Neither papillae nor wax crystals were observed on the adaxial surface of the leaf sheath (Fig. 1c), whereas on the abaxial surface of the leaf sheath, there were small papillae (approximately 1.5 µm; Fig. 1d).

A contact angle meter was used to quantify water repellency on these leaf surfaces (Fig. 1a–d bottom). Greater contact angles indicate superior water repellency. In particular, a surface exhibiting contact angles >150° is considered superhydrophobic (Barthlott et al. 2017; Koch and Barthlott 2009). Under different experimental conditions, the contact angles for rice leaves are reportedly 157 ± 2° and 162° (Barthlott and Neinhuis 1997; Neinhuis and Barthlott 1997). Under our experimental conditions, the contact angle for the adaxial leaf blade was 156°, which represents the greatest value among the 4 parts of the 6th leaf; the smallest value was for the adaxial surface of the leaf sheath (91°) (Fig. 1f, g). The values for the abaxial surface of the leaf blade and the leaf sheath were 144° and 141°, respectively (Fig. 1f).

As a representative reference species, lotus (*Nelumbo nucifera*) was also analyzed. The adaxial surface of lotus leaves had numerous papillae similar to those of rice (Fig. 1e), together with wax crystals of distinct morphology (Fig. 1e, inset). The contact angle was 158°, slightly higher than that of the rice adaxial blade (Fig. 1f, h). These results indicate that water repellency differs between regions of the rice leaf, with the adaxial surface of the blade exhibiting the greatest repellency. Furthermore, the structural organization of papillae and wax crystals in rice mirrors that of lotus, a classic model of superhydrophobicity.

### Changes in leaf surface structure during leaf development and plant growth stages

**Figure 2:**
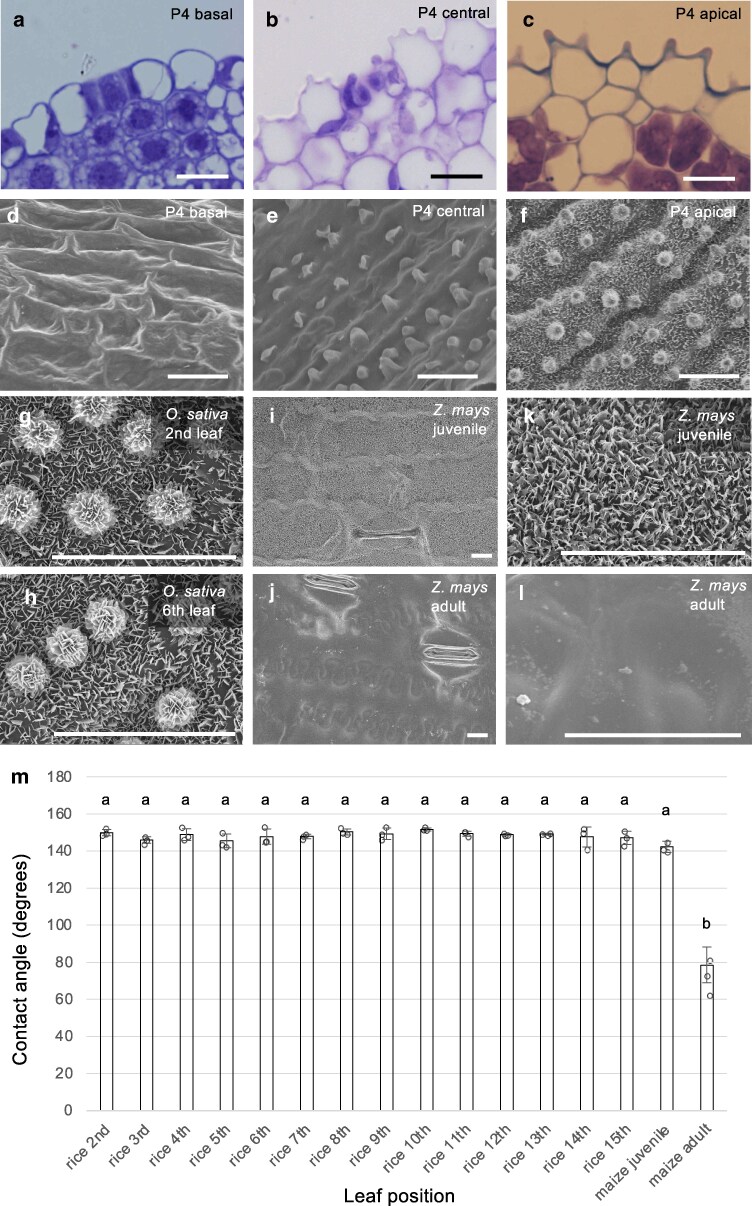
Changes in leaf surface structure during development and growth stages. a–c) Plastic sections showing epidermal structure of the adaxial surface of the sixth leaf primordium at the P4 stage. d–f) Scanning electron microscopy images of the adaxial surface of the sixth leaf primordium at the P4 stage. The basal part (a, d); the central part (b, e); the apical part (c, f). g–l) Scanning electron microscopy images of the adaxial surface of the leaf blade in rice and maize. g, h) High-magnification images of mature rice juvenile (second leaf) and adult (sixth leaf) blades. i, j) Epidermal structure of mature maize juvenile (fourth leaf) and adult (the penultimate leaf below the flag leaf) blades. k, l) Higher magnifications of (i) and (j), respectively. Note that epidermal wax crystals are visible in (g), (h), and (k) but absent in (l). Scale bars in (a-l) = 10 µm. m) Contact angles of the adaxial leaf blades in various leaves of rice and maize. Contact angles were measured at the central region of the adaxial surface of the leaf blade, avoiding the midrib. Error bars indicate standard deviation (*n* = 3). Different letters above bars indicate statistically significant differences among the leaf parts based on Tukey's HSD test (*P* < 0.05).

To investigate how leaf surface microstructures are formed during development, we first analyzed the 6th leaf primordium at the plastochron4 (P4) stage in wild-type rice plants 20 days after germination. Various parts of the leaf from the base to the apical axis were observed in plastic sections and SEM. The surface of the immature epidermal cell layer at the base of the leaf blade had no papillae or wax crystals (Fig. 2a, d). On the epidermal cells at the center of the leaf blade, there were multiple small protrusions (papillae primordia) but no wax crystals (Fig. 2b, e). On the surface of the apical part of the leaf blade, almost complete papillae and wax crystals were present. In addition, when toluidine blue was applied, the epidermal cells were clearly stained, suggesting a thickening of the cuticle here (Fig. 2c, f). Because cells at the apical tip of a primordium are developmentally older than those at the base, the observed differences along the leaf axis reflect both positional differences and relative developmental age. In addition, the P4 leaf primordium, in which these developmental stages are observed, is completely enclosed by the outer leaf (P5), suggesting that the developmental stage at which water repellency is actually required occurs at a later stage. To clarify the relationship between epidermal structures observed during leaf development and the water repellency of mature leaves, we measured contact angles at the basal, middle, and apical regions of the fully expanded 6th leaf blade. No significant differences in contact angle were detected among these regions (Figure S1a). Likewise, SEM observations revealed no obvious differences in epidermal structures, including papillae morphology and wax crystal deposition, among the 3 regions (Figure S1b). These results indicate that both water repellency and epidermal surface structures are relatively uniform along the proximal–distal axis of the mature leaf blade. Together, these observations suggest that although papillae formation and wax deposition proceed sequentially during leaf development, with papillae forming first and wax subsequently accumulating from the apical toward the basal region of the developing leaf primordium, these developmental processes are ultimately completed uniformly throughout the mature leaf blade regardless of position.

In grasses such as rice and maize, vegetative development involves a transition from the juvenile to the adult phase, which is reflected in morphological and physiological differences among leaves. In maize, it has been reported that the amount of epicuticular wax on the leaf surface decreases in adult leaves compared with juvenile leaves (Sylvester et al. 1990; Poethig 1990). In rice, early leaves such as the second leaf represent the juvenile phase, whereas later leaves such as the sixth leaf correspond to a mature vegetative stage after the juvenile-to-adult transition (Itoh et al. 2005). Therefore, in this study, we compared the second leaf (juvenile leaf) and the sixth leaf (adult leaf) to examine developmental differences in leaf surface properties. In addition, maize leaves were also analyzed for comparison. In rice, visual inspection of wax crystal deposition and the morphology of papilla by SEM revealed no noticeable differences between juvenile (the second leaf) and adult leaves (the sixth leaf) (Fig. 2g, h). In contrast, in maize, juvenile leaves (the fourth leaf) exhibited wax crystal deposition similar to that of rice, but papillae differentiation was absent (Fig. 2i, k). In adult leaves (the penultimate leaf below the flag leaf), neither papillae differentiation nor wax crystal deposition was observed (Fig. 2j, l). To examine how water repellency changes with plant developmental stage, we measured contact angles in both rice and maize. In rice, contact angles were measured on the adaxial surface of leaf blades at various leaf positions within the same individual. Contact angles were consistently around 150° across all leaf positions, with no significant differences detected (Fig. 2m). The contact angle of juvenile maize leaves was relatively high, though lower than that of rice, whereas adult maize leaves showed a much lower value, approximately 80° (Fig. 2m). These findings indicate that, whereas rice maintains high water repellency at all growth stages through the combined presence of wax crystals and papillae on the leaf surface, in maize, water repellency varies markedly depending on the growth stage, reflecting the presence or absence of wax deposition.

### Surface structure and water repellency of wetting-leaf mutants

**Figure 3:**
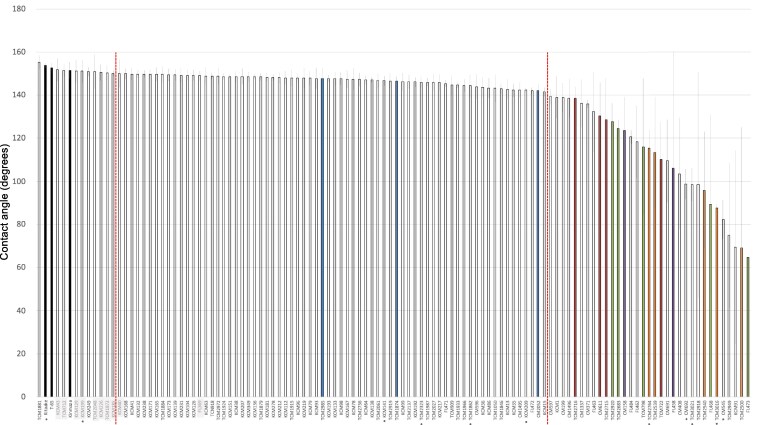
Contact angles of the adaxial leaf blades of the fourth leaf in potential mutant strains. Bars of the same color indicate mutant strains derived from mutations in the same gene. Non-filled bars indicate strains for which the causal locus has not been identified. Black: wild-type; blue: *bgl*; red: *wsl4*; green: *drp7*; purple: *wsl2*; orange: *wsl3*. Error bars indicate standard deviation (*n* ≥ 3). Data are based on 2 or more individuals, except for strains marked with an asterisk (*), which are based on a single individual. The red dotted lines separate strains with contact angles greater than or less than 150° and 140°. Strain names shown with shading indicate no statistical significance (*t*-test) at the 5% level compared with their wild-type background.

Many rice mutant strains with a wetting-leaf phenotype have been collected by the National Bioresource Project (NBRP) (Oryzabase: https://shigen.nig.ac.jp/rice/oryzabase/). These probably include mutants with reduced water repellency. To characterize these potential mutants, we selected 113 strains that were described as having a wetting-leaf phenotype from the rice mutant stock maintained by the NBRP. In total, 116 lines (113 mutants plus 3 wild-type cultivars: T-65, Kinmaze, and Kitaake, which serve as the backgrounds of the mutants) were analyzed. We measured contact angles on the adaxial surface of the 4th leaf blade. When the mean values of the contact angles of these 113 strains were examined, most of the strains exhibited smaller contact angles than the wild-type cultivars. These strains could be separated into 2 groups. In 1 group, the contact angle fell below the superhydrophobicity threshold of 150°, but the reduction was within approximately 10° and little variation was observed among individuals (140° < contact angle < 150°; Fig. 3). In contrast, in the other group, the contact angle was markedly reduced and exhibited substantial variation among individuals (contact angle < 140°; Fig. 3).

**Figure 4:**
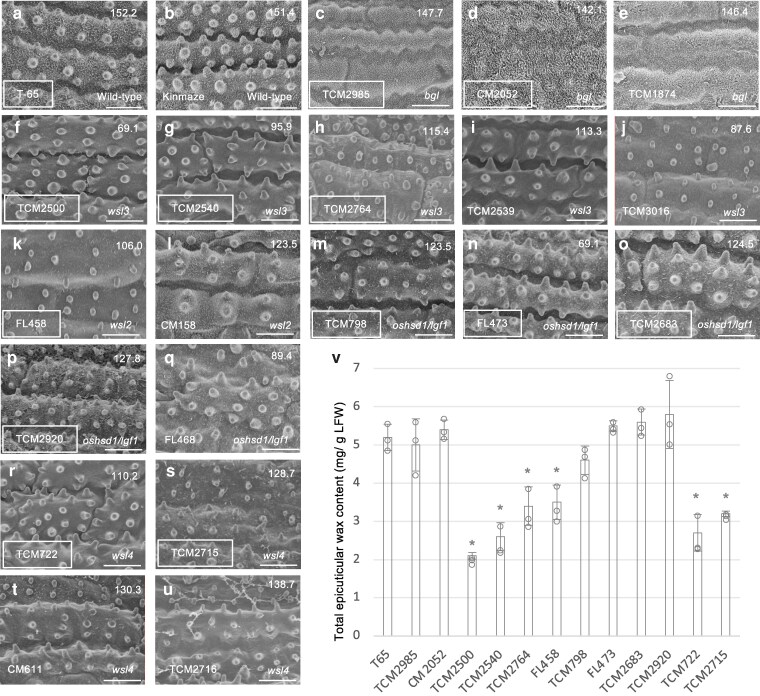
Scanning electron microscopy images showing the epidermal structure of wild types and 19 wetting-leaf mutants. a, b) Wild-type. c–e) The first type of epidermal structure: papillae absent and normal wax crystals. f–u) The second type of epidermal structure: normal papillae but reduced or abnormal wax crystals. For each panel, the contact angle (top right), strain name (bottom left), and mutant gene (bottom right) are indicated. Strain names highlighted with a white square denote strains whose epicuticular wax content was measured in panel (v). Scale bars = 10 µm. v) Total epicuticular wax content of wild type (T-65) and representative mutant strains. The vertical axis shows the amount of wax (mg) per fresh weight (g) of mature leaf (LFW). These values should be interpreted primarily as relative comparisons among genotypes rather than absolute measures of wax deposition per unit leaf area. Error bars represent the standard deviation of 3 independent samples. Asterisks (*) above the bars indicate statistical significance at the 5% level (*t*-test) compared with the wild type (T-65).

We screened the potential wetting-leaf mutants using SEM and identified 19 strains with abnormal leaf surface microstructure (Fig. 4). These were separated into 2 types. The first type of mutant had no papillae but normal epicuticular wax on the leaf surface (Fig. 4c–e). The second type had normal papillae but reduced or abnormal epicuticular wax crystals (Fig. 4f–u). The first group of mutants included the 3 strains with contact angles >140°. The second group of mutants all had contact angles of 140° or less, with the smallest contact angle being only 65° (Fig. 3). To test whether SEM observations reflected actual wax load, epicuticular wax content was quantified in wild-type plants and representative mutants. Wax content was expressed as mg wax per g fresh leaf weight rather than as an absolute amount per unit leaf area and was used primarily for relative comparisons among genotypes. Mutants in which wax crystals were nearly absent (eg TCM2500, TCM722) had markedly reduced wax content, whereas mutants with no papilla (eg TCM2985, TCM2052) and with abnormal but still detectable wax crystals (eg TCM2683, TCM2920) did not show significant changes (Fig. 4v). These results indicate that reduced water repellency in rice wetting-leaf mutants arises from 2 distinct abnormalities: loss of papillae or altered quantity/quality of wax crystals. Among these, wax quantity had the stronger effect, because wax/papillae-free mutants still had very high contact angles (ie >140°; Fig. 4c, e) while waxless mutants with papillae had the lowest contact angles (ie < 100°; Fig. 4f, g). Wax quality also seems to be important since we observed that the FL473 mutant, rich in wax with papillae but different wax crystal micromorphology, had the lowest contact angle (69.1°; Fig. 4n). It seems that all 3 characteristics, namely high wax content, normal wax micromorphology, and papillae, are needed to achieve superhydrophobicity, ie contact angles above 150° (Fig. 4a, b).

### Identification of the genes responsible for reduced water repellency in leaves

**Figure 5:**
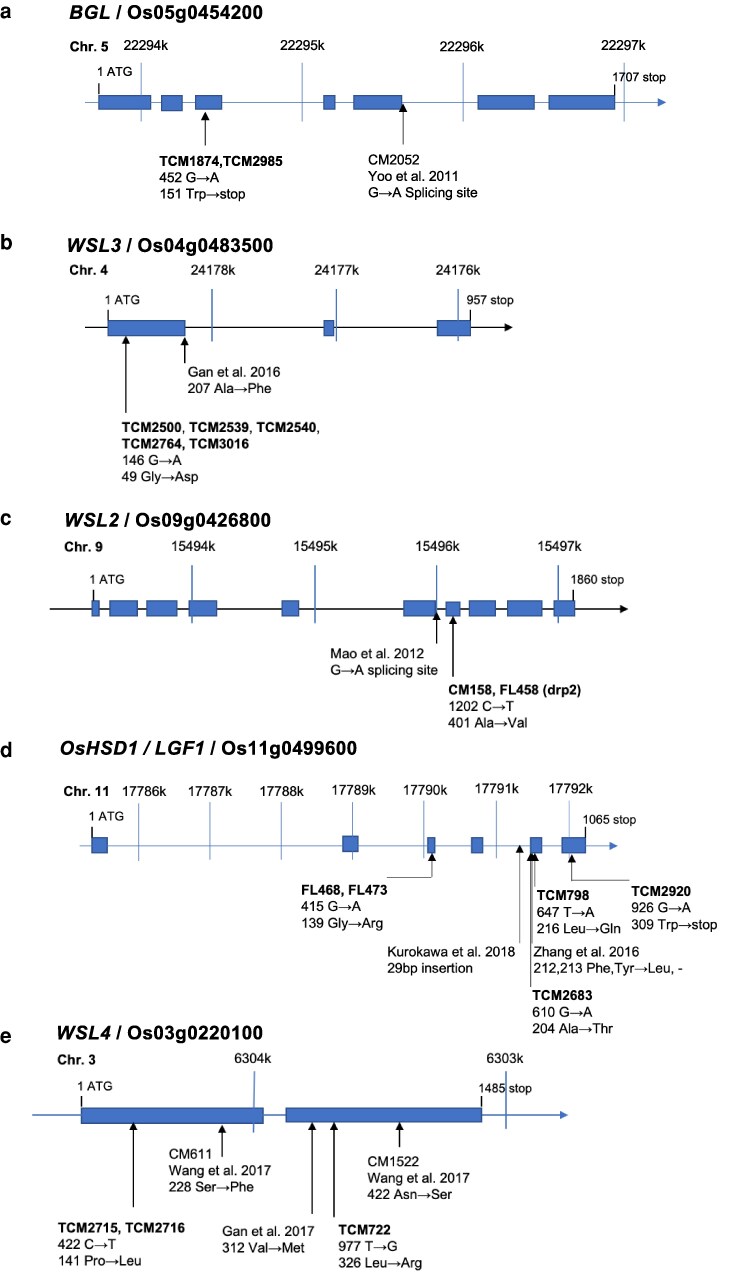
Genes and mutations involved in leaf water repellency. Mutant strains and mutations in *BGL*/Os05g0454200 (a), *WSL3*/Os04g0483500 (b), *WSL2*/Os09g0426800 (c), *OsHSD1/LGF1*/Os11g0499600 (d), and *WSL4*/Os03g0220100 (e). The mutant strains in bold are those identified in this study. The type and position of each mutation is shown, together with the resulting change in amino acid.

While investigating mutations associated with reduced water repellency, we discovered that 1 of the 19 mutants, CM2052, had already been described as *bright green leaf* (*bgl*) mutant KL208, which exhibits altered leaf color and has no small epidermal papillae (Yoo et al. 2011). Therefore, we predicted that the mutations in TCM1874 and TCM2985 were also in the *BGL* gene, because these mutants also had no small epidermal papillae (Fig. 4c, e). We determined the DNA sequence of the *BGL* gene in both mutants and found that the same mutation generated a stop codon in the second exon in each case (Fig. 5a, FigureS2). This strongly suggests that TCM1874, CM2052, and TCM2985 all contained mutant alleles of the *BGL* gene.

Of the remaining 16 strains, CM611 reportedly has a mutation in the *WSL4* gene, which encodes a KCS (Wang et al. 2017; Fig. 5e). Of the remaining 15 strains, 9 were crossed with the wild-type Indica variety Kasalath, and the F_2_ population was used to map the corresponding mutations. The mutations were mapped to the long arm of chromosome 4 (TCM2500, TCM2539, TCM2540, TCM2764, and TCM3016), the long arm of chromosome 9 (CM158 and FL458), the long arm of chromosome 11 (TCM2920), and the short arm of chromosome 3 (TCM2716) (Fig. 5, Table S1). The following genes that are involved in wax biosynthesis are located in these 4 chromosomal regions: *WSL3* (Gan et al. 2016), *OsGL1–1*/*WSL2* (Qin et al. 2011; Mao et al. 2012), *OsHSD1/LGF1* (Zhang et al. 2016; Kurokawa et al. 2018), and *WSL4* (Gan et al. 2017; Wang et al. 2017). Therefore, we determined the DNA sequences of these genes in all 15 mutant strains and identified single nucleotide substitutions in 1 of the 4 genes in all strains (Fig. 5b–e, FigureS2). The reduced or absent accumulation of epidermal wax crystal phenotypes was very similar to those of mutants that had already been characterized (Qin et al. 2011; Mao et al. 2012; Gan et al. 2016, 2017; Zhang et al. 2016; Wang et al. 2017; Kurokawa et al. 2018), strongly suggesting that the 15 mutant strains with abnormal epicuticular wax were derived from mutations in 4 genes involved in wax biosynthesis.

The relationship between the mutations and the reductions in contact angle was analyzed. For mutant alleles of *BGL*, the contact angles of TCM1874, TCM2985, and CM2052 were 146.4°, 147.7°, and 142.1°, respectively (Fig. 3). Among the mutants of wax biosynthesis-related genes, mutations in *WSL3* that produced the same amino acid substitution were observed in TCM2500, TCM2539, TCM2540, TCM2764, and TCM3016 (Fig. 5b). However, the contact angles differed significantly from 69.1° in TCM2500 to 115.4° in TCM2764 (Fig. 3). Similar results were observed for the *WSL2* gene, in which the same mutation caused an amino acid substitution but the contact angle in CM158 was 123.5° but 106.0° in FL458 (Fig. 3 and 5c). Four different mutations in 5 mutant strains and 2 mutations in 3 mutant strains were found in *OsHSD1/LGF1* and *WSL4*, respectively, but all of these mutations produced amino acid substitutions (Fig. 5d, e). Because most identified mutations were amino acid substitutions rather than null alleles, their effects may vary in severity. To further evaluate functional importance, we aligned the mutated residues with orthologs from other Poaceae species and found that all substitutions occur at conserved sites (Figure S3), suggesting that they are likely to be functionally significant. Overall, there appeared to be no association between the type of wax biosynthesis gene and the magnitude of the effect on contact angle: the magnitude of contact angle reduction could vary among mutant alleles of any of the 4 genes.

Identification of genes responsible for wetting-leaf phenotypes revealed that water repellency is regulated by at least 1 gene controlling papillae differentiation and 4 genes involved in the biosynthesis of epicuticular wax.

### Expression of wax biosynthesis-related genes

**Figure 6:**
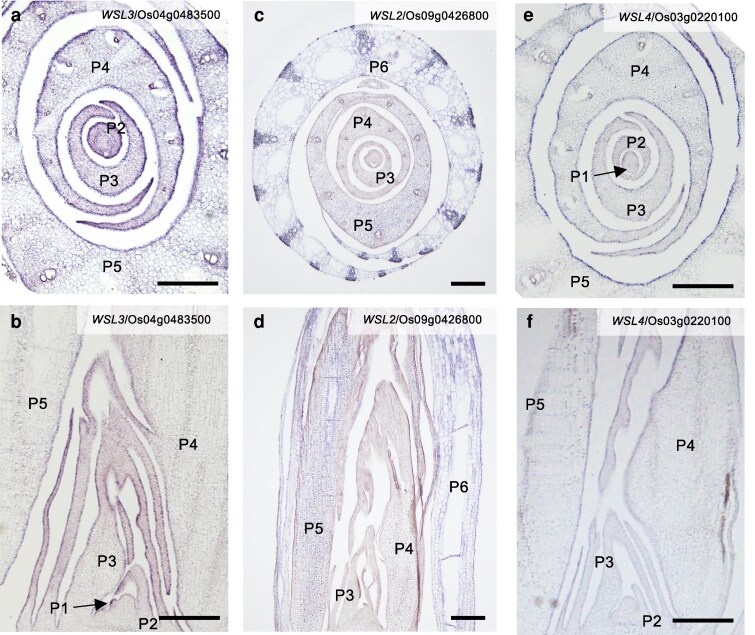
Spatial expression pattern of wax biosynthesis-related genes highlighted by in situ hybridization. Expression of *WSL3*/Os04g0483500 (a and b), *WSL2*/Os09g0426800 (c and d), and *WSL4*/Os03g0220100 (e and f). Cross sections and longitudinal sections of wild-type seedlings 25 days after germination. The leaf stage (Px) is labeled. Scale bars = 200 µm.

The expression patterns of the 4 wax biosynthesis genes (*WSL3*, *WSL2*, *OsHSD1/LGF1*, and *WSL4*) were investigated using the β-glucuronidase reporter gene, and staining corresponding to all 4 genes was observed in mature leaves (Qin et al. 2011; Mao et al. 2012; Gan et al. 2016, 2017; Zhang et al. 2016; Wang et al. 2017). However, epicuticular wax accumulation begins in leaf primordia at P4 (Fig. 2) and information regarding tissue specificity and the timing of expression is necessary to understand the regulation of epidermal wax accumulation. Contrary to our expectations, in situ hybridization using immature shoot tissue revealed expression of *WSL3* and *WSL4* (encoding a KCR and KCS, respectively) early in leaf development (Fig. 6a, b, e, f). *WSL3* and *WSL4* expression was detected not only in the L1 layer of P1 to P5 leaf primordia but also in the SAM. However, the expression pattern of *WSL2* differed from the other genes and a signal was observed at P5 and P6, as well as in vascular bundles and sclerenchyma (Fig. 6c, d). The *OsHSD1/LGF1* signal was too weak to provide a reliable expression pattern.

Next, to determine whether the expression of these genes was affected by deficiencies in wax biosynthesis or papillae formation, expression levels were analyzed by qRT-PCR in each mutant (Figure S4). The expression of *WSL2* was reduced in the *wsl3* mutant, but there were no significant changes in the expression of any of the other genes in any of the mutants. These results suggest that the 4 genes involved in the biosynthesis of cuticular wax have different expression patterns, although the corresponding mutants show similar abnormalities in leaf wax accumulation. In addition, the expression level of each gene is largely unaffected by mutations in the other genes.

### Diversity of water repellency among rice varieties

To investigate diversity among rice cultivars and related species, we evaluated water repellency and epidermal structure in cultivated rice and wild *Oryza* species. First, we measured the contact angles of 55 accessions from the WRC, which includes cultivated rice and genetically diverse accessions maintained by NARO Genebank (https://www.gene.affrc.go.jp/databases-core_collections_wr_en.php; (Tanaka et al. 2020; Table S2). More than half of the accessions had contact angles exceeding 150°, and the smallest contact angle was 143° (WRC3), indicating that most of these cultivated rice accessions showed superhydrophobicity (Figure S5). Observations of leaf surface structure using low-vacuum SEM showed that all of the accessions were similar (Figure S6). This suggests that superhydrophobicity is conserved among rice cultivars and is an essential trait for rice cultivation around the world.

**Figure 7:**
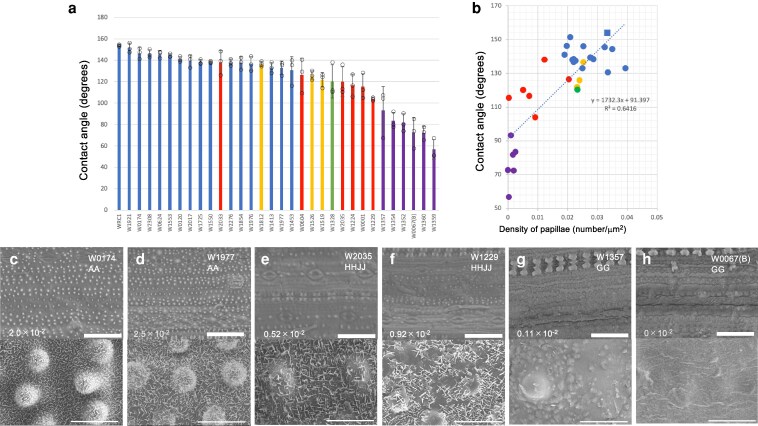
Water repellency, epidermal structure, and genome type in wild *Oryza* species. a) Contact angles of 31 wild *Oryza* accessions selected by shower tests and WRC1 (Nipponbare) as a representative of cultivated rice. Bars of the same color indicate the same genome type. Blue: AA; orange: CC; red: HHJJ; green: BBCC; purple: GG. Error bars indicate standard deviation (*n* = 3). b) Correlation between contact angle and papillae density. Plots of the same color indicate the same genome type, as described above. The square represents WRC1. c–h) The epidermal feature of several wild *Oryza* accessions revealed by scanning electron microscopy. For the upper part of each panel, accession names and genome types are indicated in the top right corner and papillae density (papillae/µm^2^) is shown in the bottom left corner. Scale bars = 50 µm. The bottom part of each panel represents the structure and density of wax crystals. Scale bars = 5 µm.

Next, to understand the origins of superhydrophobicity in cultivated rice, we investigated water repellency in wild *Oryza* accessions maintained by the National Institute of Genetics, Japan (https://shigen.nig.ac.jp/rice/oryzabase/locale/change?lang=en). To identify accessions with reduced water repellency, we used a qualitative “shower test” screen, in which water was showered onto leaves of approximately 400 wild *Oryza* accessions and retention of droplets was noted. This was followed by quantitative contact angle measurements on positive accessions. A total of 31 “shower test positive” accessions were selected, and we sampled mature leaf blades from these accessions to compare with WRC1 (Nipponbare) as a representative of cultivated rice (Table S3). Unlike the WRC results, the contact angles of selected wild *Oryza* accessions suggested diverse degrees of water repellency: 25 of the 31 accessions had contact angles below 140°, which was significantly lower than the contact angles observed in cultivated species (Fig. 7a). Observations of leaf surface structure using SEM revealed that several accessions with small contact angles had significantly lower papillae densities than the other accessions (Fig. 7c–h, Figure S7). Then, we investigated the relationship between the papillae density and the contact angles in the leaves of 32 accessions (Fig. 7b). As a result, a correlation was observed between contact angle and papilla density. However, when compared among individual lines, papilla density did not necessarily account for the differences in contact angle, suggesting that factors beyond papilla density influence repellency.

The selected accessions of wild *Oryza* species could be classified into the following genome types: 15 AA (13 *O*. *rufipogon* and 2 *O*.*longistaminata*), 3 CC (2 *O*. *eichingeri* and 1 *O*. *rhizomatis*), 1 BBCC (*O*. *minuta*), 6 HHJJ (4 *O*. *ridleyi* and 2 *O*. *longiglumis*), and 6 GG (5 *O*. *meyeriana* and 1 *O*. *granulata*) species. The shower test–positive AA genome accessions had mean contact angles of approximately 140° and tended to have greater papillae densities than other genome types. On the other hand, HHJJ genome accessions had smaller contact angles and lower papillae densities, whereas the BBCC and CC accessions were phenotypically intermediate. Unlike other semi-aquatic species, the GG genome species that inhabit shady forests had extremely small contact angles and low papillae densities, and W0067(B) had almost no papillae (Fig. 7h). To further investigate the diversity of leaf surface structures, we compared wax morphology in 2 different HHJJ and GG species (Fig. 7c–h). Both HHJJ accessions displayed wax crystals similar in form to AA accessions but at lower density (Fig. 7e, f), whereas the 2 GG accessions lacked discernible wax crystals altogether (Fig. 7g, h).

Together, these results indicate that most wild *Oryza* species with the AA genome, which are the closest relatives of cultivated rice, are inferred to exhibit high water repellency due to dense papillae and abundant wax crystals. In contrast, certain lineages such as the GG species display very low repellency associated with both reduced papilla density and the absence of wax crystals. These findings suggest that quantitative variation in papilla density and wax abundance, rather than qualitative structural differences, underlies interspecific variation in water repellency across the genus.

## Discussion

There are 4 different epidermal regions in rice leaves: the adaxial/abaxial leaf blade and the adaxial/abaxial leaf sheath (Itoh et al. 2005). Of these 4 regions, water repellency was greatest on the adaxial surface of the leaf blade and poorest on the adaxial surface of the leaf sheath. The poor water repellency of the latter compared to the other 3 regions is most likely due to differences in epidermal structure. The adaxial surface of the leaf sheath is the only region that had neither papillae nor wax crystals. It is also the only region where the subsequent leaf remains in close contact, and it may not need a water-repellent structure because it interacts less with the external environment. In addition, the adaxial surface of the leaf blade resembles that of lotus, suggesting that superhydrophobicity in rice is achieved through the same structural basis as in lotus.

In maize, epidermal wax accumulates on the surface of lower leaves but is not present on higher leaves. This is considered one of the hallmarks of the juvenile-to-adult phase change in maize (Poethig 1990). In the present study, we found that the developmental variation in wax accumulation in maize directly influences leaf water repellency (Fig. 2). In rice, however, neither contact angle nor wax deposition varied significantly with leaf position under our growth conditions (Fig. 2). In contrast, maize shows developmental regulation of repellency, rice maintains strong water repellency throughout its life cycle, likely reflecting adaptation to its semi-aquatic environment.

An analysis of water repellency and surface structure in wetting-leaf mutants in cultivated rice backgrounds revealed 2 types of mutant with different characteristics: 1 with normal epicuticular wax but lacking papillae and 1 with normal papillae but deficient in the accumulation of epicuticular wax. The first type of mutant was derived from mutations in the *BGL* gene, with *bgl* mutants previously characterized as luminous green plants that lack small papillae on their leaves (Yoo et al. 2011). We found that *BGL* is also involved in water repellency. The reduction in water repellency, which was associated with a reduction in contact angle of approximately 10° due to the loss of small papillae, was clearly smaller than that due to wax abnormalities. However, this change is significant for the physical properties of the leaf surface. A superhydrophobic surface is defined as 1 with a static contact angle greater than 150°, thereby creating a self-cleaning effect (Barthlott et al. 2017; Koch and Barthlott 2009). If the surface of a rice leaf has normal epicuticular wax but lacks papillae, as in the *bgl* mutants, the contact angle cannot exceed 150°, resulting in the loss of superhydrophobicity. Therefore, at least under the genetic backgrounds and experimental conditions examined in this study, papillae differentiation appears necessary for rice leaves to achieve the superhydrophobic threshold (>150° static contact angle). Only a few grass species have papillae on their leaf surfaces, and those that do are not necessarily closely related phylogenetically (Prasad et al. 2011). Consequently, rice probably acquired or maintained papilla formation during the evolution of the *Poaceae*, fostering superhydrophobic characteristics suitable for semi-aquatic environments. How papillae differentiate remains unclear, but papillae protrusions occur by the P4 stage of leaf development (Fig. 2) together with other changes in epidermal cells. Understanding the molecular mechanisms involved in this process may provide opportunities to manipulate water repellency by modifying surface structures.

The second type of mutant with reduced water repellency was derived from mutations in 4 wax synthesis genes. One characteristic of wax-deficient mutants was the large variation in contact angle among individual plants. We interpret this as partly a measurement issue: at high contact angles, droplets are stably supported by the structured waxy surface, whereas at lower contact angles, droplets spread more readily and are more sensitive to micro-heterogeneity on the leaf surface. In wax-deficient mutants, patchy or irregular coverage of epicuticular wax crystals likely further contributes to this variability. Thus, reduced water repellency may amplify both biological heterogeneity and technical noise, leading to higher variation in contact angle values when surfaces are no longer uniformly superhydrophobic. In addition to the variation in contact angle among individual plants, mean angles varied among the mutant strains. Perhaps the effects on water repellency vary depending on gene function and the strength of mutant alleles. In our analysis, however, most of the mutations in the 4 wax-synthesis genes produced changes in the amino acids, and there were no obvious null alleles. Therefore, we were unable to quantify the contribution of each gene to water repellency. In addition, even mutant strains with the same mutation showed variation in mean contact angle. Perhaps the differences between mutant strains are influenced by differences between individual plants and background mutations in the mutant strains. Notably, in the mutant carrying a defect in *OsHSD1/LGF1*, which is required for C30 primary alcohol synthesis (Kurokawa et al. 2018), abnormal and reduced wax crystals were observed despite no substantial reduction in total wax content (Fig. 4m–p, v). This observation, consistent with previous reports (Zhang et al. 2016; Kurokawa et al. 2018), indicates that water repellency depends not only on the amount of epicuticular wax but also on its chemical composition and structural organization. Thus, both quantitative and qualitative properties of the wax layer jointly determine the degree of leaf hydrophobicity. In this study, epicuticular wax content was expressed relative to leaf fresh weight rather than leaf surface area. Therefore, the wax quantification presented here should primarily be interpreted as a relative comparison among genotypes rather than an absolute measure of wax deposition per unit leaf area.

We investigated the expression of 4 genes involved in epidermal wax synthesis and found that *WSL3* and *WSL4*, which are part of the FAE complex, are expressed in epidermal cells during early leaf development, including in the L1 layer of the SAM. This expression pattern was similar to that of *ONI1* and *ONI2*, which belong to the same KCS gene family as *WSL4* (Ito et al. 2011; Tsuda et al. 2013; Gan et al. 2017; Wang et al. 2017). *oni1* and *oni2* mutants were seedling lethal and showed developmental abnormalities including leaf fusion due to loss of function of the L1 layer (Ito et al. 2011; Tsuda et al. 2013). In contrast to *oni1* and *oni2*, all *wsl4* mutants were viable and no abnormalities were observed during early development. Although different KCS enzymes may have specific substrates (Batsale et al. 2021), *WSL4* may exhibit functional redundancy with *ONI1/ONI2* during early leaf development. Alternatively, it may have functions other than epidermal wax synthesis (Gan et al. 2017). Interestingly, 2 genes involved in modification after VLCFA biosynthesis showed expression patterns that differed from those of *WSL3* and *WSL4*. Expression of *WSL2* was observed not only in the epidermis but also in the primordia of inner leaves (Qin et al. 2011). This suggests that *WSL2* functions not only in epicuticular wax synthesis but also in inner leaf tissues (Qin et al. 2011). In addition, mutations in each of 4 wax synthesis genes and in papilla-forming genes did not affect the expression of the wax synthesis genes. Therefore, under our experimental conditions, the expression of these 4 genes was regulated independently of changes in wax synthesis and papilla formation, although feedback regulation does reportedly occur among the wax synthesis genes (Mao et al. 2012). In this study, physicochemical measurements and structural observations (contact angle measurements and SEM analyses) were conducted using plants grown under greenhouse conditions that closely resemble normal cultivation environments. In contrast, gene expression analyses (in situ hybridization and qRT-PCR) were performed using plants grown under controlled growth chamber conditions to ensure reproducible developmental staging of young shoot tissues. Because environmental conditions can influence epidermal development and wax biosynthesis, the expression patterns described here should be interpreted primarily as indicative of spatiotemporal expression during early leaf development rather than as a direct quantitative correspondence with the physicochemical properties measured in greenhouse-grown plants.

No significant differences in contact angle or surface structure were observed among cultivated rice varieties from the WRC. This suggests that water repellency is conserved in cultivated rice. Among the WRC accessions, WRC51 is an upland rice cultivar but exhibits a similar degree of water repellency to that of other paddy rice accessions. Thus, water repellency may also be advantageous for varieties grown in dry conditions. On the other hand, analyses of wild *Oryza* accessions revealed substantial variation in both leaf surface structure and the degree of water repellency among accessions. Interestingly, among the wild *Oryza* accessions examined in this study, AA genome accessions including *O*. *rufipogon*, thought to be ancestral to cultivated rice (*O*. *sativa*) (Huang et al. 2012), tended to be more water repellent and had higher densities of small papillae. Because the population we used in our shower test screen contained more than 200 *O*. *rufipogon* accessions, many of the shower test-negative accessions probably had very good water-repellent properties. Therefore, leaf superhydrophobicity was probably not selected for during the domestication of rice but was already present in the ancestral species and inherited by *O*. *sativa*. In contrast, some HHJJ genome species exhibited moderate reductions in both papillae density and wax accumulation. The GG genome species are the only group adapted to forests rather than semi-aquatic environments (Shi et al. 2020), showed extremely low papilla density, and lacked detectable wax crystals. These observations indicate that papilla differentiation and wax deposition are likely adaptive traits for *Oryza* species in semi-aquatic habitats. Furthermore, although a correlation between contact angle and papillae density was observed among the wild *Oryza* accessions analyzed, comparisons among individuals indicated that differences in papillae density alone could not fully account for variation in contact angle (Fig. 7b). Together, these results suggest that quantitative variation in wax deposition and papilla density, rather than qualitative structural differences, explains the diversity of water repellency among wild *Oryza* species. On the other hand, the adaptive significance discussed here is inferred from the distribution of leaf surface traits among cultivated rice and wild *Oryza* species together with previously reported ecological functions of leaf water repellency. Because fitness-related traits such as gas-film maintenance, submergence tolerance, pathogen attachment, self-cleaning efficiency, and field performance were not directly evaluated in this study, the adaptive implications of these traits remain to be tested experimentally.

Many studies have sought to predict what surface structures are important for water repellency, including superhydrophobicity (Neinhuis and Barthlott 1997; Koch and Barthlott 2009; Bhushan 2012; Barthlott et al. 2017). However, there are few experimental reports describing how changes in surface structure affect water repellency in a single species or in closely related species. Our study reveals part of the structural, genetic, and adaptive basis for superhydrophobicity in rice leaves. Our analyses suggest that water repellency in rice is more strongly influenced by the quantity and quality of epicuticular wax than by papillae density. Although a multivariate approach would be valuable for quantitatively assessing the relative contributions of wax amount, wax morphology, and papillae density to water repellency, the present dataset does not permit rigorous partitioning of these effects because these traits covary among genotypes and species. To rigorously evaluate the contribution of each factor, it will be necessary to analyze larger datasets under a common genetic background, using more quantitative assessments of surface traits and lines in which individual traits are altered independently. Future studies using genetically defined materials will be necessary to determine the extent to which each structural and biochemical trait contributes to water repellency and superhydrophobicity.

From an applied perspective, rice leaf superhydrophobicity is important for both crop resilience and agricultural management, as modulation of water repellency can influence leaf wetness duration, gas-film maintenance under flooding, and the retention and uptake of foliar-applied nutrients and pesticides (Taylor 2011; Kurokawa et al. 2018; Jolliffe et al. 2023). Beyond these practical implications, our findings highlight the biological significance of leaf-surface architecture in mediating plant–environment interactions through the regulation of water dynamics at the leaf surface. By identifying genetic determinants that independently control wax quantity and micromorphology as well as papilla formation, this study provides a foundation for modifying leaf-surface traits to suit different agricultural environments and managements, not only in rice but also in other crop and plant species, and may further inform biomimetic applications inspired by natural superhydrophobic surfaces.

## Materials and methods

### Plant materials

Taichung 65 (T-65) was used as a representative variety of rice (*Oryza sativa* L.). In addition, Kinmaze and Kitaake were included because they are the backgrounds of several wetting-leaf mutants analyzed in this study. These varieties belong to the japonica subspecies, which is mainly cultivated in temperate East Asia. Kasalath is widely used as a parent strain for positional cloning; it belongs to the aus subspecies, which is cultivated primarily in South Asia. Maize leaves were sampled from B73 plants obtained from the NARO Genebank in Japan and grown under natural conditions. Lotus leaves were taken from a representative accession maintained at the Institute for Sustainable Agro-ecosystem Services, The University of Tokyo. Wetting-leaf mutants in rice derived from N-methyl-N-nitrosourea-treated accessions stored at Kyushu University were identified by keyword-based “text screening” in Oryzabase (http://www.shigen.nig.ac.jp/rice/oryzabase/), in which strains are listed with phenotype descriptions; the keyword “wetting leaves” was used to obtain candidates. There was a total of 113 strains, including mutant strains from the T-65, Kinmaze, and Kitaake background lines (TCM, CM, and KCM) and marker gene accumulation lines (FL).

For diversity analysis of rice cultivars, 55 of the 69 accessions from the World Rice Core Collection (WRC) stored at the Genebank for Agricultural Biological Resources of the National Agriculture and Food Research Organization (NARO) were used (Table S2). The WRC collection covers 90% of allelic diversity (Kojima et al. 2005; Tanaka et al. 2020; https://www.gene.affrc.go.jp/databases-core_collections_wr.php).

For diversity analysis of wild *Oryza* species, we used 31 accessions from the wild *Oryza* species collection (https://shigen.nig.ac.jp/rice/oryzabase/strain/wildCore/list) stored and maintained at the National Institute of Genetics in Japan (Table S3).

### Growth conditions

The plants used for measuring contact angles and observing surface structures were grown under standard cultivation conditions for rice in Tokyo: seeds were sown in late April, and plants were cultivated in a greenhouse under natural day length and ambient seasonal temperatures (uncontrolled). Plants were watered regularly to maintain soil moisture in paddy-style conditions. Humidity was not artificially controlled and therefore reflected natural greenhouse conditions. For in situ hybridization and quantitative real-time polymerase chain reaction (qRT-PCR), wild-type and mutant seeds were sterilized and sown on soil or Petri dishes and subsequently grown in a growth chamber under a 16 h light (30 °C)/8 h dark (25 °C) photoperiod with warm-white LED lamps (200 μmol m^−2^ s^−1^).

Wild *Oryza* species grown under natural conditions in a greenhouse or a field at the National Institute of Genetics in Mishima were screened using qualitative “shower test” in early July, in which water was sprayed onto leaves and droplet retention was visually scored. Leaf blades that had developed and emerged completely from the leaf sheath but were not senescent were sampled for quantitative contact angle measurements and scanning electron microscopy (SEM) analysis.

### Measurement of contact angles

The center of the leaf blade or leaf sheath was cut to an appropriate size, fixed on a glass slide using double-sided tape with the surface facing upward, and placed on the stage of a contact angle meter (LSE-ME3 Nick, Saitama, Japan). A syringe was used to apply 1 µL of water to the leaf surface, and the contact angle was measured for 5 s. Droplets were placed on representative flat regions of the central portion of the leaf parts, avoiding the midrib. Leaf positions were counted from the bottom up, with the first leaf emerging from the coleoptile designated as the 1st leaf. For Figs 1 and 2, 7, S1, and S5, 3 separate plants were measured (*n* = 3). For Fig. 3, 3 or more measurements were performed per strain, but the number of individuals varied depending on availability: most strains had 2 or 3 plants measured, while in a few cases measurements were possible on a single individual; these cases are indicated in the figure legend. The adaxial surface of the center of the leaf blade was measured 3 times per leaf.

### Observation of surface structure

Sampled leaf blades were immersed in 4% paraformaldehyde solution followed by overnight fixation. After a graded series of tert-butyl alcohol washes, samples were immersed in 100% tert-butyl alcohol and frozen at −20 °C. The frozen samples were freeze-dried using a lyophilizer (JFD-320; JEOL, Ltd, Tokyo, Japan). In addition, for selected observations of wax morphology (Fig. 2 and Fig. 7), we also employed a direct SEM method without fixation, which better preserves wax crystals.

Samples were prepared for SEM under a stereomicroscope and secured to the stage using carbon tape. A platinum ion coating was applied for 90 s using an E-1030 ion sputter coater (E-1030; Hitachi, Tokyo, Japan). Leaf surface structure was observed via SEM (S-4800; Hitachi). To view the surface structure of WRC lines and wild *Oryza* species, we applied low-vacuum tabletop SEM using the Miniscope TM1000 (Hitachi) and JCM-7000 NeoScope instruments (JEOL). Sampled leaf blades were glued to the stage using carbon tape under a stereomicroscope, and leaf surface structure was observed without further treatment.

### Measurement of total epicuticular wax content

SEM observations of leaf surface microstructures were performed using younger leaves as described in the corresponding sections. In contrast, wax quantification requires a relatively large amount of tissue, and therefore, these measurements were conducted using fully expanded mature leaves. Mature leaf blades were collected from 3 independent plants grown under identical natural conditions in a concrete-bordered paddy field, sampling approximately 15 cm from the penultimate leaf below the flag leaf. Each leaf was stored in a tube to prevent desiccation, and leaf fresh weight was measured immediately prior to analysis. Accurate measurement of leaf surface area is technically difficult in rice because detached leaf blades undergo rapid deformation and curling during drying. Therefore, epicuticular wax content was normalized to leaf fresh weight rather than leaf surface area. This sampling and measurement strategy ensured that comparable leaf tissues were analyzed across different genotypes. Total cuticular wax was quantified using a colorimetric method modified from Ebercon et al. (1977). For wax extraction, leaf blades with fresh weight recorded were cut into ∼3 cm segments and immersed for 10 s in 5 mL of chloroform preheated to 55 °C. After complete evaporation of the chloroform, 1 mL of acidic K_2_Cr_2_O_7_ solution, prepared according to Ebercon et al. (1977), was added to the cuticular wax deposited at the bottom of the tube. The mixture was incubated at 100 °C with agitation for 30 min. After cooling, 2.4 mL of water was added, and optical density at 590 nm was measured using a spectrophotometer. The obtained optical density was normalized to the fresh weight of the extracted leaves, using the relationship between wax weight and optical density determined in wild-type rice leaves as a standard, and expressed as mg wax content per g fresh weight of leaf tissue.

### Preparation of plastic sections

Leaf samples were cut under a stereomicroscope, immersed in 4% (w/v) paraformaldehyde and 1% Triton X-100 in 0.1 M sodium phosphate buffer, and fixed overnight. The fixed samples were sequentially washed with ethanol (30%, 50%, 70%, 90%, and 100%) and then immersed in Technovit 7100:100% ethanol (1:1) and 100% Technovit 7100 (Heraeus Kulzer, Hanau, Germany). Samples were hardened by adding Technovit 7100:Hardener II (15:1). Embedded samples were cut into sections 3 µm thick using a microtome. Sectioned samples were stained with 0.05% toluidine blue and observed under an optical microscope.

### Identification of genes responsible for the wetting-leaf phenotype

To map TCM2500, TCM2540, TCM2764, TCM3016, CM158, FL458, TCM2920, and TCM2716, individual plants exhibiting the wetting-leaf phenotype were selected from an F_2_ population after crossing each mutant with Kasalath. DNA from selected individuals was analyzed for linkage using simple sequence repeat and cleaved amplified polymorphic sequence mapping markers (Table S4). The chromosomal regions identified by these markers were analyzed for genes involved in wax biosynthesis or creating epidermal structure to identify candidate genes. DNA sequences and mutations for 5 candidate genes from the mutant strains were determined by Sanger sequencing.

### *In situ* hybridization

*In situ* hybridization was performed using antisense RNA probes. To synthesize the probes, cDNA fragments from 2 genes (*WSL2* and *WSL4*) were amplified and cloned using the Zero Blunt TOPO PCR Cloning kit (Invitrogen, Carlsbad, CA, United States; Table S5). *WSL3* cDNA was obtained from a plasmid containing full-length cDNA (AK060512) provided by the National Institute of Agrobiological Sciences, Japan. These cDNA plasmids were used as templates, and antisense RNA probes were synthesized using the DIG RNA Labeling kit (Roche Diagnostics GmbH, Mannheim, Germany). Sense RNA probes for each gene were used as negative controls.

Samples of T-65 plants were fixed using 4% (w/v) paraformaldehyde and 1% Triton X-100 in 0.1 M sodium phosphate buffer for 48 h at 4 °C. Then they were dehydrated using a graded series of ethanol washes, immersed in 1-butanol, and embedded in Paraplast Plus (McCormick Scientific, Berkeley, MO, United States). The samples were cut into sections 8 µm thick using a rotary microtome, and longitudinal and cross-sections containing shoot apical meristem (SAM) tissue were prepared. Hybridization and chromogenic reactions were performed as described by (Miya et al. 2025). After staining, the sections were mounted using Poly Mount (Polysciences, Inc., Warrington, PA, United States) and observed under a light microscope.

### qRT-PCR

We analyzed the expression levels of the *WSL3*, *WSL2*, *OsHSD1/LGF1*, and *WSL4* genes. Wild-type (T-65 and Kinmaze) and mutant (CM611 for *wsl4*, TCM2500 for *wsl3*, CM158 for *wsl2*, TCM798 for *oshsd/lgf1*, and TCM2985 for *bgl*) seeds were sterilized and sown on wet filter papers in Petri dishes. Whole shoots from 2 seedlings at 2 days after sowing were collected for each sample. Samples were frozen, and RNA was extracted using TRIzol reagent (Invitrogen). The extracted RNA was treated with recombinant DNase I (TaKaRa Bio, Inc., Shiga, Japan), and cDNA was synthesized using the High-Capacity cDNA Reverse Transcription kit (Life Technologies, Carlsbad, CA, United States). qRT-PCR was performed using the StepOne Real-Time PCR system (Life Technologies) with TaqMan Fast Universal PCR Master Mix and FAM-labeled TaqMan probes for each gene. The *OsRAD6* gene was used as an internal standard. The TaqMan probes and primers used for each gene are listed in Table S6. For all experiments, we analyzed 3 technical and 4 biological replicates.

### Measurement of papillae density

Images of leaf surfaces obtained using the tabletop Miniscope TM1000 (Hitachi) and JCM-7000 NeoScope SEM instruments were imported into ImageJ software (National Institutes of Health, Bethesda, MD, United States) to measure papillae densities. Densities were measured from 3 separate samples as the number of papillae in a predefined area.

## Supplementary Material

kiag514_Supplementary_Data

## Data Availability

The data that support the findings of this study are available from the corresponding author upon reasonable request.
