# Supplementary material for: Structural, genetic, and adaptive basis of superhydrophobicity in rice leaves
Source: Plant Physiol. 2026 Jul 23;201(3):kiag514. doi: 10.1093/plphys/kiag514 (PMC13413574; doi:10.1093/plphys/kiag514)
Supplement: kiag514_Supplementary_Data [file kiag514_supplementary_data.zip › SFigures_R3.pdf]

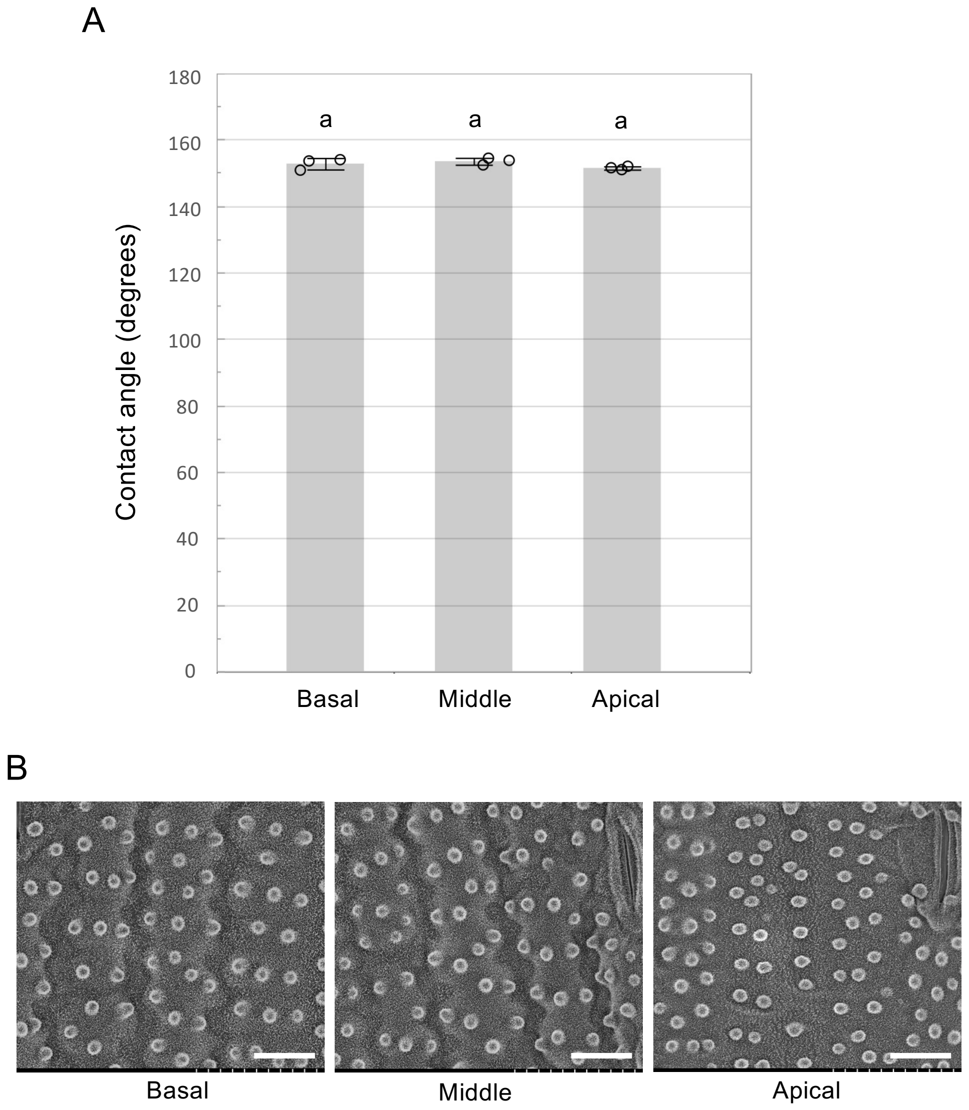

Supplementary Figure S1. Water repellency and surface structure along the fully expanded sixth leaf blade. Contact angles (**A**) and scanning electron micrographs of surface structures (**B**) at the basal, middle, and apical regions of the adaxial surface of the sixth leaf blade. The leaf blade was divided into three equal sections along the proximal–distal axis, and measurements and observations were performed at the center of each section, avoiding the midrib. Bars represent mean ± SD (n = 3). The same letter indicates no significant difference according to Tukey's HSD test ($P < 0.05$) (A). Scale bars = 10 μm (B).

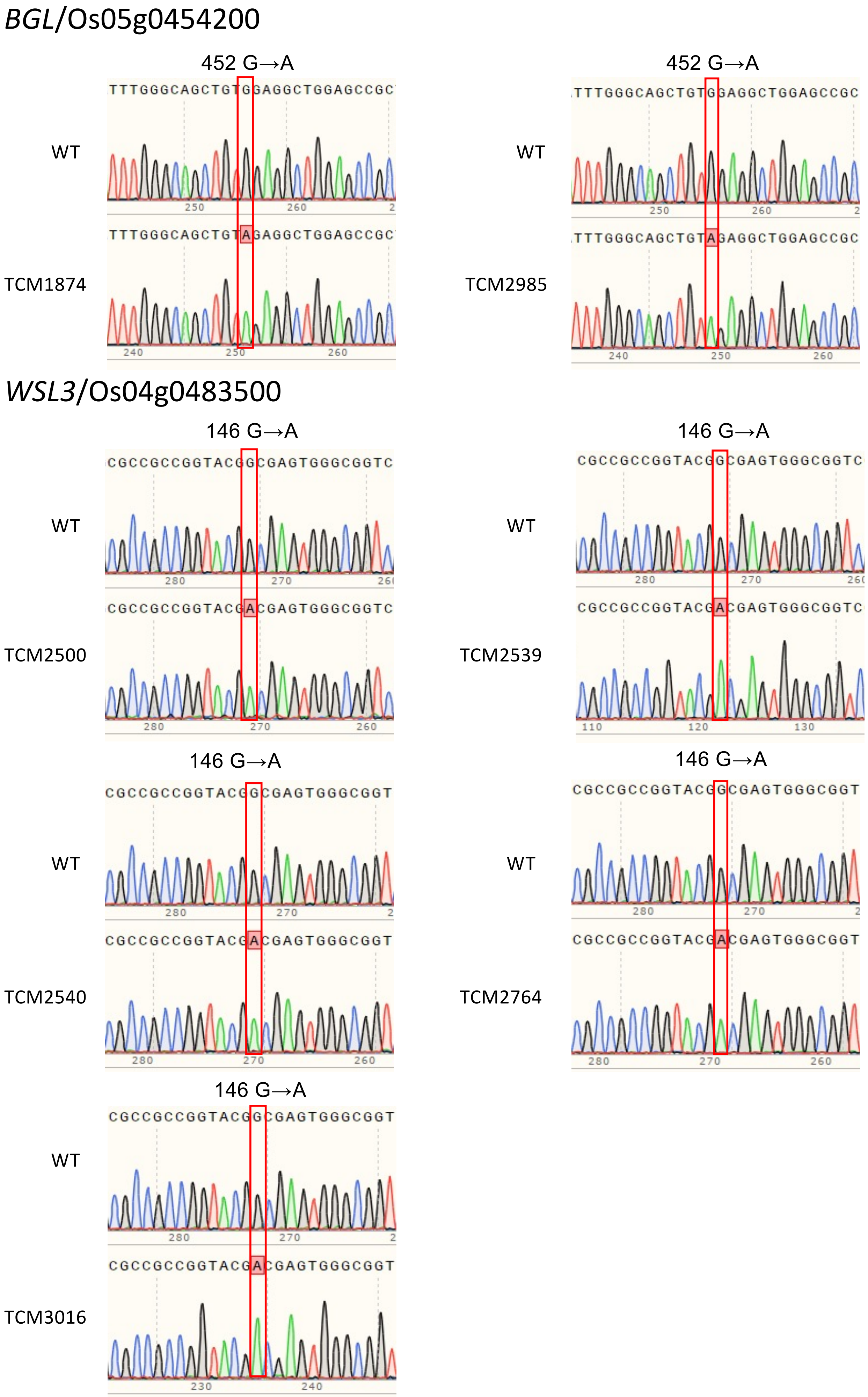

Supplementary Figure S2. Mutations identified in five genes in wetting-leaf mutants. Upper panels: wild type; lower panels: mutants. Red boxes indicate the mutated nucleotides.

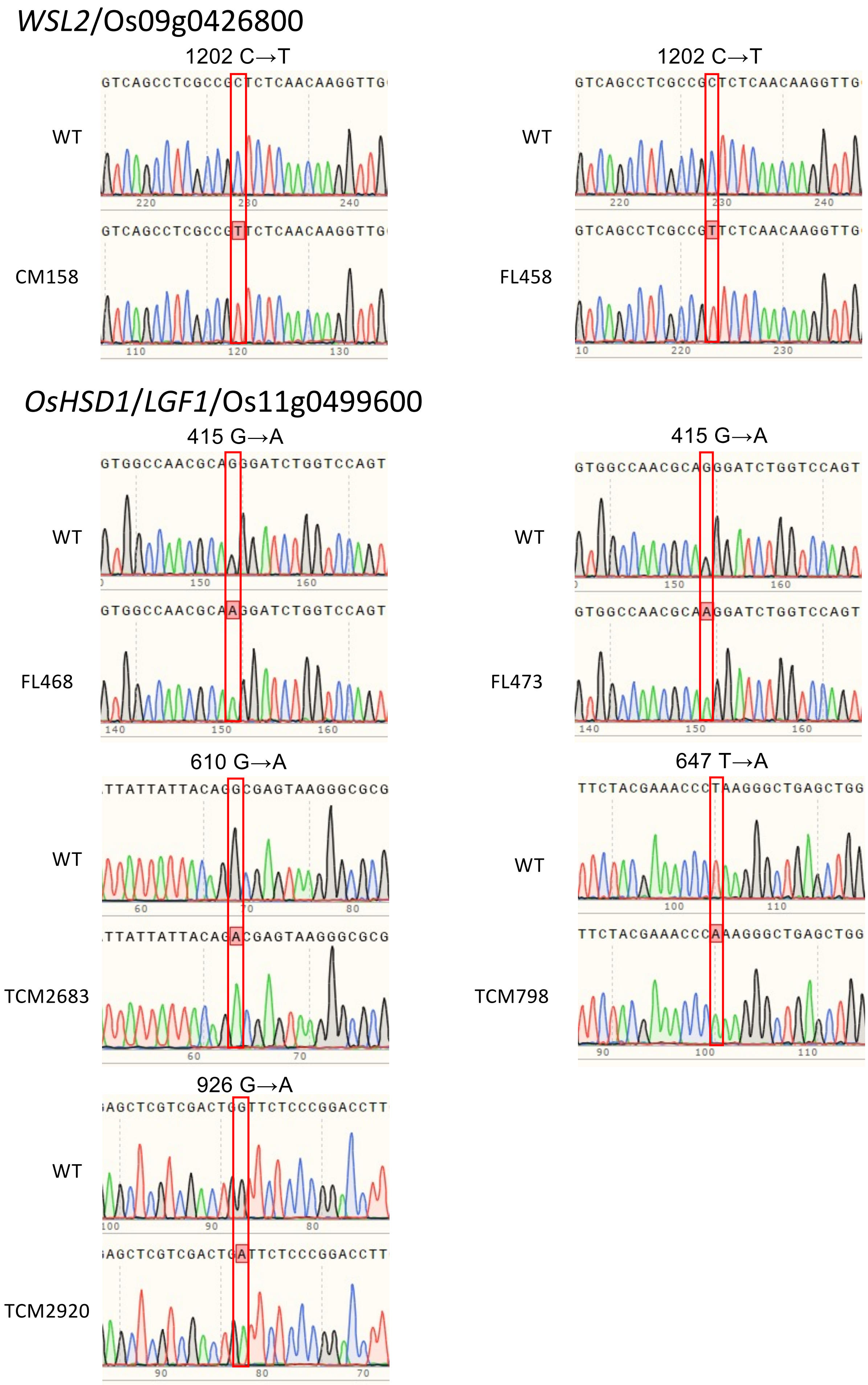

Supplementary Figure S2 (continued). Mutations identified in five genes in wetting-leaf mutants. Upper panels: wild type; lower panels: mutants. Red boxes indicate the mutated nucleotides.

WSL4/Os03g0220100

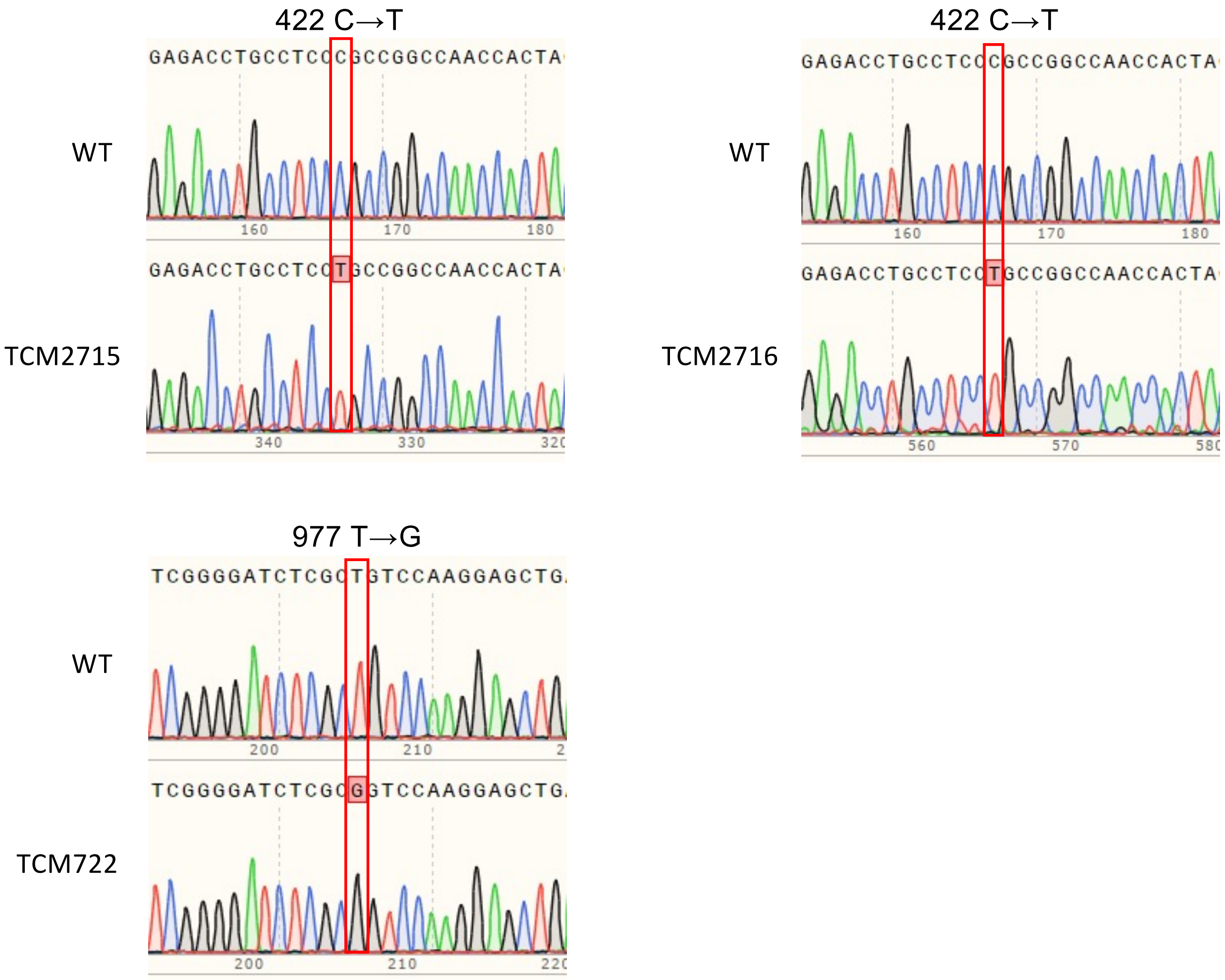

Supplementary Figure S2 (continued). Mutations identified in five genes in wetting-leaf mutants. Upper panels: wild type; lower panels: mutants. Red boxes indicate the mutated nucleotides.

A

```
XP_002439905.1 [Sorghum bicolor]              MARGSTGAGE----EEEEERAASEALTADSADEE--GRRGSSSSASSEAASS-TVSYTYS     53
NP_001131743.2 [Zea mays]                     ---------------MARGDTASEALTADSADEE--GRRGSSSSASSDAASSFSCTYSPA     43
Os05g0454200/BGL [Oryza sativa]               MARGGGGGEEAAEEE----REVSEALTADSSA-DEECRRGSSSSSAS---SGDASSESYC     52
XP_010231312.1 [Brachypodium distachyon]      MARGGGGPAEE---EEEGEMAVSETLTAESADDEERRRRGSSGSSAS---SEAASSESYC     54
XP_044337236.1 [Triticum aestivum]            MARGGGGGAAEEEEEEEEGMAASDTLTAESEE----CRRG-SSSSAS---SVAASTDSYC     52
XP_044978624.1 [Hordeum vulgare]              MARG-GGAAAEEEEEEEEEMAVSETLTAESEE----CRRG-SSSSAS---SVAASSESYC     51
                                                                .*::***:*       *** *.*::*   *  : : : .

XP_002439905.1 [Sorghum bicolor]              PPDEWQQKVAIKTCVSVVSADVAVAGAGGAD-----DG-----GNEKKPPRASEMEMMKE     103
NP_001131743.2 [Zea mays]                     PPDEWHKVAIIKTCVSVSADAAG--GGGGKD-----QKPRGVVAAADRHGGASEMEMMKE     96
Os05g0454200/BGL [Oryza sativa]               PPDEWQKV-AIKTCVSSDLVVVSAEPAKEKKPPPPPSSPRVDAAPADKHHRPSEMEMMKE     111
XP_010231312.1 [Brachypodium distachyon]      PPDEWQQAVAIKTCVTDDATGK--------AK----PPDAKEKLPGAEKHRAPELELMKE     102
XP_044337236.1 [Triticum aestivum]            PPDEWQQA-AIKTCVTDDAVAA--------IAKANPPAPGKEIPPLAERHRAPEVELMKE     103
XP_044978624.1 [Hordeum vulgare]              PPDEWQQV-AIKTCVTDDAAAK---------PPAPAPAPGKENPPGAERHRAPEVELMKE     101
                                              *****::   *****:                                .     *:*:***

XP_002439905.1 [Sorghum bicolor]              RFSKLLLGEDMSGSGKGVCTALAISNAITNLCATIFGQLWRLEPLLPEKKAMWRREMDWL     163
NP_001131743.2 [Zea mays]                     RFSKLLLGEDMSGSGKGVCTALAISNAVTNLCATVFGQLWRLEPLPPEKKAMWRRELDCL     156
Os05g0454200/BGL [Oryza sativa]               RFAKLLLGEDMSGGGKGVCTALAISNAITNLCATIFGQLWRLEPLLPEKKTMWRREMDWL     171
XP_010231312.1 [Brachypodium distachyon]      RFAKLLLGEDMSGSGKGVCTALAISNAITNLCATIFGQLWRLEPLAPEKKAMWRREMDWL     162
XP_044337236.1 [Triticum aestivum]            RFSKLLLGEDMSGSGKGVCTALAISNAITNLCATIFGQLWRLEPLLPEKKAMWRREMDWL     163
XP_044978624.1 [Hordeum vulgare]              RFSKLLLGEDMSGSGKGVCTALAISNAITNLCATIFGQLWRLEPLLPEKKAMWRREMDWL     161
                                              **:**********.*************:******:********* ****:*****:* *
```

TCM1874,TCM2985
151 Trp→stop

```
XP_002439905.1 [Sorghum bicolor]              LCVSDHIVELVPTWQTFPDGTRLEIMTSRPRSDLYINLPALRKLDNMLLEILEGFRDAEF     223
NP_001131743.2 [Zea mays]                     LCVSDHIVELVPTWQTFPDGTRLEIMTSRPRSDLYINLPALRKLDNMLLEILEGFRDVEF     216
Os05g0454200/BGL [Oryza sativa]               LCVSDHIVELVPTWQTFPDGSKLEIMTSRPRSDLYINLPALRKLDHMLLEILESFRDPEF     231
XP_010231312.1 [Brachypodium distachyon]      LCVSDHIVELVPTWQTFPDGTRLEIMTSRPRSDLYINLPALRKLDNMLLEILESFRDTEF     222
XP_044337236.1 [Triticum aestivum]            LCVSDHIVELVPTWQTFPDGTRLEIMTSRPRSDLYISLPALRKLDHMLLETLESFRDTEF     223
XP_044978624.1 [Hordeum vulgare]              LCVSDHIVELVPTWQTFPDGTRLEIMTSRPRSDLYINLPALRKLDHMLLETLESFRDTEF     221
                                              ********************::**************.********:**** **.*** **

XP_002439905.1 [Sorghum bicolor]              WYVDQGICAPDCDGSASYRR-TFHRRDDKWWLPVPRVPHGGLCEATRRQVEHRRDCANQI     282
NP_001131743.2 [Zea mays]                     WYVDQGICAPDCDSSASYRR-TFHRRDDKWWLPVPHVPHGGLREATRRQLEHRRDCASQI     275
Os05g0454200/BGL [Oryza sativa]               WYVDQGICPPDRDGSAPFML-TFHRRDEKWWLPVPRVPPGGVGETTRRQLEHKRDCASQI     290
XP_010231312.1 [Brachypodium distachyon]      WYADQGIC----DGAASFRKSSFHRRDEKWWLPVPRVSLGGLGEATRRQLEHKRDCANQI     278
XP_044337236.1 [Triticum aestivum]            WYLDQGICAPDCDGSASFRR-PAHRRDEKWWLPVPRVPPGGLRDATRRQLEHKRDAANQI     282
XP_044978624.1 [Hordeum vulgare]              WYVDQGICAPDCDGSASFRR-PAHRRDEKWWLPVPRLPPGGLRDATRRQLEHKRDAANQI     280
                                              ** *****    *.:* :     ****:*******::  **: ::****:**:**.*.**

XP_002439905.1 [Sorghum bicolor]              LKAAMAINSNALAEMDVPDSYLDSLPKNGRATLGDVIYRYITSDHFSPDCLLDCLDLSSE     342
NP_001131743.2 [Zea mays]                     LKAAMAINSNALAEMAVPVSYLDSLPKNGRAALGDVIYRYITSDQFSQDYLLDCLDLSSE     335
Os05g0454200/BGL [Oryza sativa]               LKAAMAINSNALAEMDVPDSYLDSLPKNGRATLGDIIYRYITSDQFSPDCLLDCLDLSSE     350
XP_010231312.1 [Brachypodium distachyon]      LKAAMAINSNALAEMDVPESYHDSLPKNGRATLGDIIYRYITSEQFSPDCLLDCLDLSSE     338
XP_044337236.1 [Triticum aestivum]            LKAAMAINSNALAEMDVPDSYHDSLPKNGRATLGDIIYRYITSEQFSPDCLLDCLDLSSE     342
XP_044978624.1 [Hordeum vulgare]              LKAAMAINSNALAEMDVPDSYHDSLPKNGRATLGDIIYRYITSEQFSPDCLLDCLDLSSE     340
                                              *************** ** ** *********:***:*******::** * **********

XP_002439905.1 [Sorghum bicolor]              YQALEIANRVEASVYVWRRRGGGGHGAAAKPASRAGAKSSWGIVKDMIMDTEKR-DLLA     401
NP_001131743.2 [Zea mays]                     YQALEIANRVEASIYVWRRKGGGG-------------ARSSWGIVKDMVMDTEKR-DLLA     381
Os05g0454200/BGL [Oryza sativa]               YQALEIANRVEASIYVWRRRGTSGA-------ASRAGNKSSWGIVKDMIMDTEKRDDLLA     403
XP_010231312.1 [Brachypodium distachyon]      YQAVEIANRVEASIYVWRRRGTAAG--------KSAGGKSSWGMVKDMIMDTEKRGDLLA     390
XP_044337236.1 [Triticum aestivum]            YQAVEIANRVEAAVYVWRRRGTAAA--------KSAGTKSSWGMVKDMIMDTEKRGDLLA     394
XP_044978624.1 [Hordeum vulgare]              YQAVEIANRVEAAIYVWRRRGAA-A--------KSVGTKSSWGMVKDMIMDTEKRGDLLA     391
                                              ***:********::*****:* .                :****:****:****** ****

XP_002439905.1 [Sorghum bicolor]              ERAEGLLISLKQRFPGLTQTSLDMSKIQYNKDVGKSILESYSRVLESLASNIIARIDDLL     461
NP_001131743.2 [Zea mays]                     ERAEALLMSLKQRFPGLTQTSLDMSKIQYNKDVGKSILESYSRVLESLASNIIARIDDLL     441
Os05g0454200/BGL [Oryza sativa]               DRAEGLLMCLKQRFPGLTQTSLDTSKIQYNKDVGKSILESYSRVLESLASNIIARIDDLL     463
XP_010231312.1 [Brachypodium distachyon]      ERAEGLLISLRQRFPGLTQTSLDMSKIQYNKDVGKSILESYSRVLESLASNIIARIDDLL     450
XP_044337236.1 [Triticum aestivum]            ERAEGLLISLKQRFPGLTQTSLDMSKIQYNKDVGKSILESYSRVMESLASNIIARIDDLL     454
XP_044978624.1 [Hordeum vulgare]              DRAEGLLISLKQRFPGLTQTSLDMSKIQYNKDVGKSILEGYSRVLESLASNIIARIDDLL     451
                                              :***.**:.*:************ **************.****:**************

XP_002439905.1 [Sorghum bicolor]              SVDELSKQ-SDSIPSAG--VDAKIVCKINSSSSSSSKQAATVVPASGTPYATAYTTPSFS     518
NP_001131743.2 [Zea mays]                     NVDELSKQPADSIPSAG--ADAKVACKSNGS------KQATAVPASGTPYATAYATPSFS     493
Os05g0454200/BGL [Oryza sativa]               YVDERSRQAELL-PTAG-AGSGKISCMPAMSASSVPAYPVVSTSGTPPPYATAYATPSFS     521
XP_010231312.1 [Brachypodium distachyon]      HVDELSKQPDQV-P-----ADGKFIARK--AAAMVAPAP----ASGTP-YLTAYATPSFS     497
XP_044337236.1 [Triticum aestivum]            YVDELSKQADQK-LPAGVAEDGKIACKN-KKAAATAPSP----AYAVPASGTTYVTPSFS     508
XP_044978624.1 [Hordeum vulgare]              YVDELSKRTDQK-LPAGVADDGKIACNKNKKAAAMAPSP----AYPVPVSGTPYVTPSFS     506
                                               *** *::             ..*. .                        * *.*****

XP_002439905.1 [Sorghum bicolor]              PAQLSSPSKIGRALLVDRRSHHVKGPAGATKRTTARSVADRAGVAEVVKGIVVGS--AVF     576
NP_001131743.2 [Zea mays]                     PAQLSSPSKIGRALLVDRRSHHVAGAKRATA-----SAADRAGV-EVVKGMVVDGSSAIF     547
Os05g0454200/BGL [Oryza sativa]               PAQLSSPSNIGRALLVDRRSHDGRAFD----------------GSMEFMGMAVSN--AVF     563
XP_010231312.1 [Brachypodium distachyon]      PALLSSPSKIGRALLVDRRSHHHSKN-----------------GVEVVKGMLASS--RLA     538
XP_044337236.1 [Triticum aestivum]            PAQLSSPSKIGRALLVDRRAHHGRASKRS--------ALADHGGGPEVKGMLVTS--PVF     558
XP_044978624.1 [Hordeum vulgare]              PAQLSSPSKIGRALLVDRRAHHGRASKRS--------ALADHAGGPEVKGMLVSS--AVF     556
                                              ** *****:**********:*.                         . *: . .    :

XP_002439905.1 [Sorghum bicolor]              DV-PTAVTAEM 586
NP_001131743.2 [Zea mays]                     DIIPIAVPAKI 558
Os05g0454200/BGL [Oryza sativa]               DLPGL------ 568
XP_010231312.1 [Brachypodium distachyon]      AEL-------- 541
XP_044337236.1 [Triticum aestivum]            DAPLGTEL--- 566
XP_044978624.1 [Hordeum vulgare]              DAPLDTEL--- 564
```

Supplementary Figure S3. Amino acid conservation of five proteins identified in this study as being involved in water repellency across the Poaceae. Amino acids highlighted with red boxes indicate the positions altered by gene mutations identified in this study. A) BGL, B) WSL3, C) WSL2, D) OsHSD1/LGF1, E) WSL4.

B

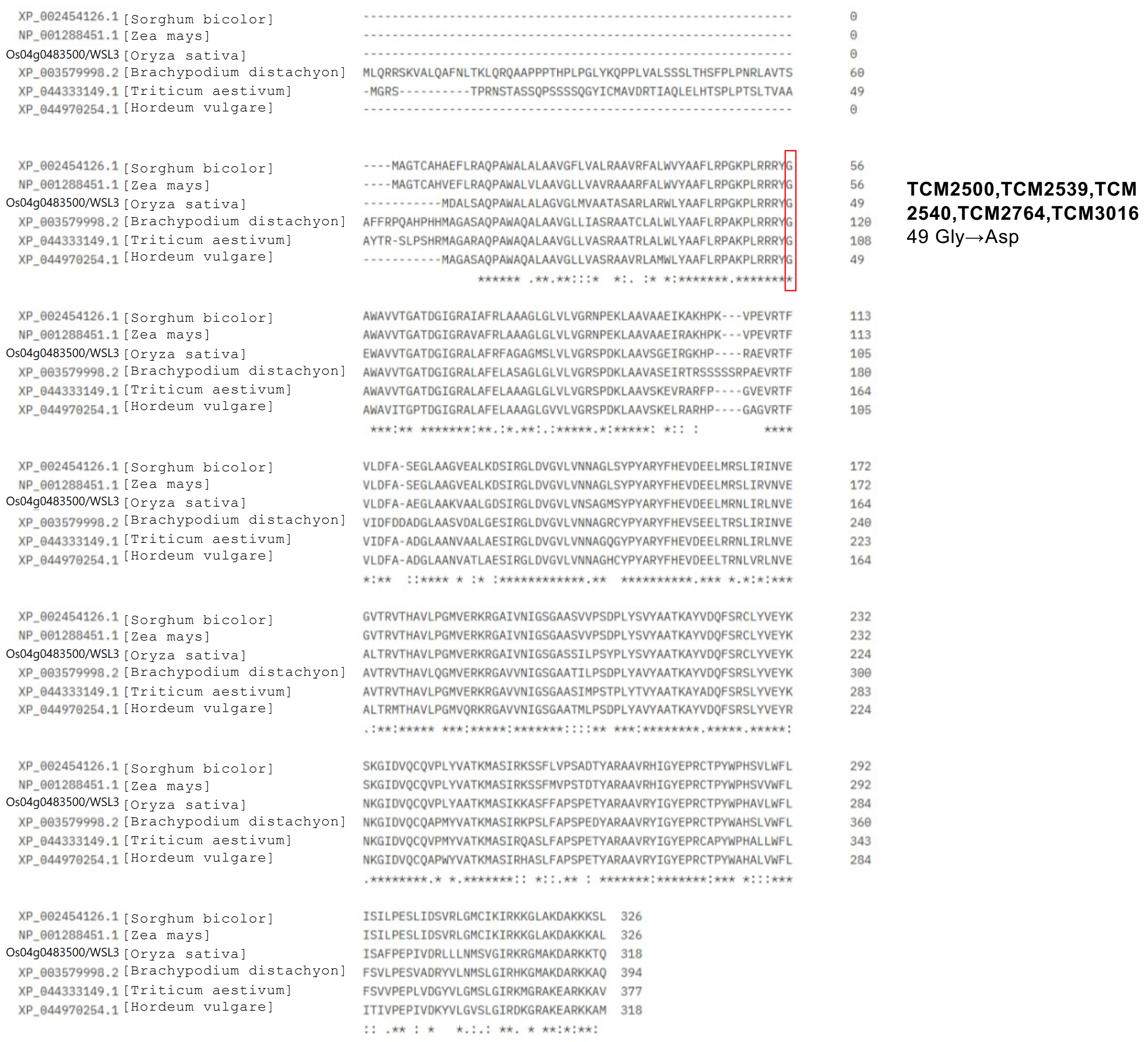

Supplementary Figure S3 (continued). Amino acid conservation of five proteins identified in this study as being involved in water repellency across the Poaceae. Amino acids highlighted with red boxes indicate the positions altered by gene mutations identified in this study. A) BGL, B) WSL3, C) WSL2, D) OsHSD1/LGF1, E) WSL4.

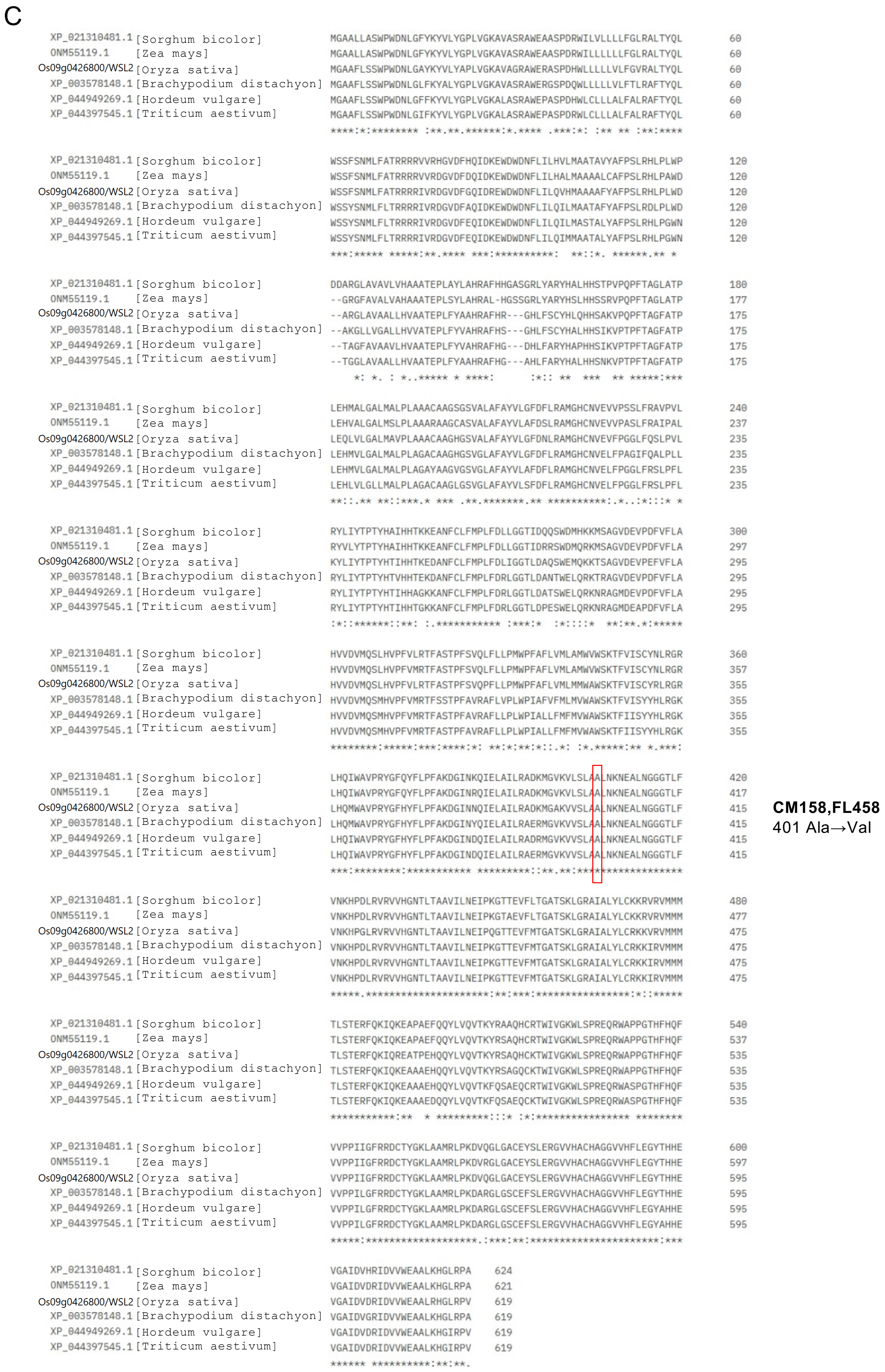

Supplementary Figure S3 (continued). Amino acid conservation of five proteins identified in this study as being involved in water repellency across the Poaceae. Amino acids highlighted with red boxes indicate the positions altered by gene mutations identified in this study. A) BGL, B) WSL3, C) WSL2, D) OsHSD1/LGF1, E) WSL4.

D

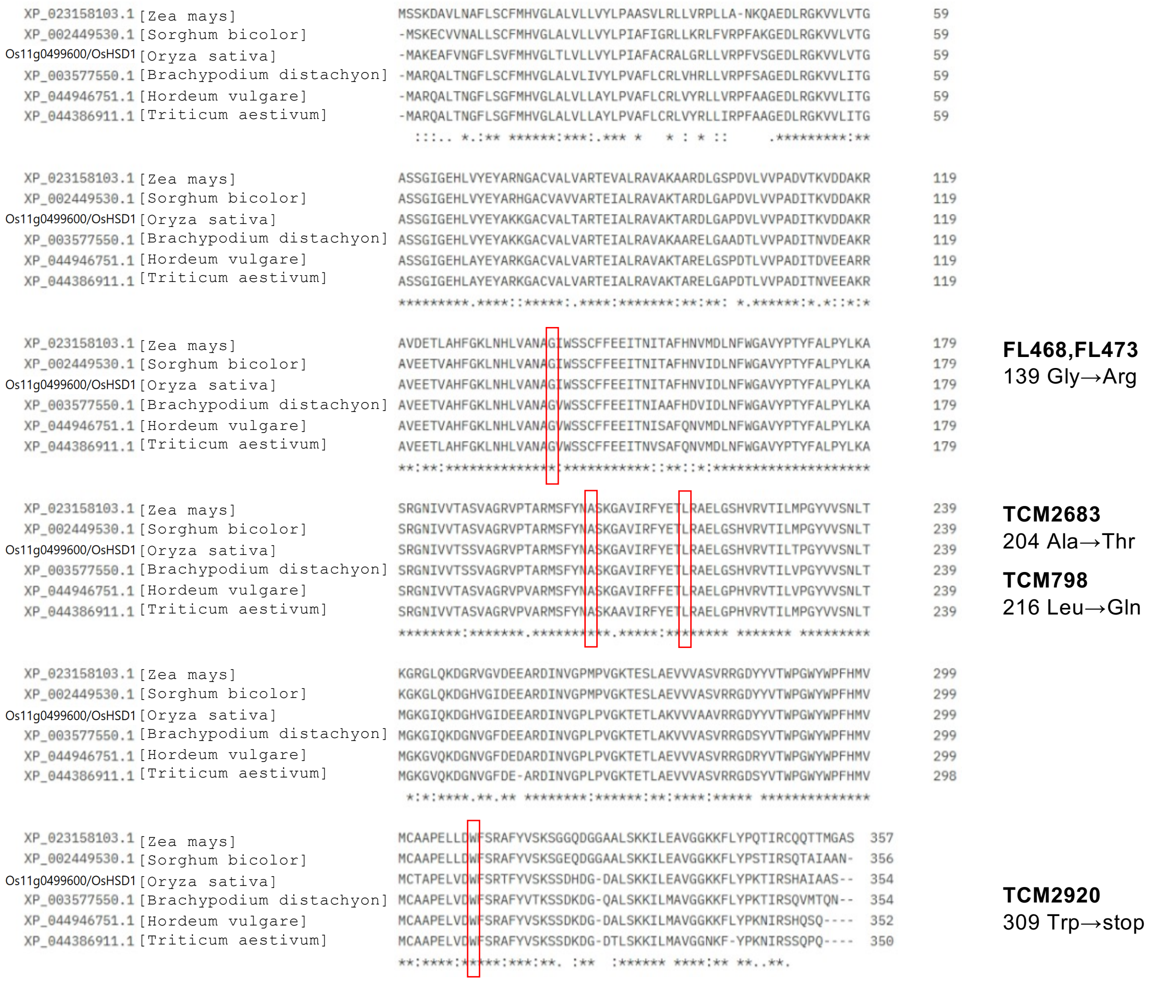

Supplementary Figure S3 (continued). Amino acid conservation of five proteins identified in this study as being involved in water repellency across the Poaceae. Amino acids highlighted with red boxes indicate the positions altered by gene mutations identified in this study. A) BGL, B) WSL3, C) WSL2, D) OsHSD1/LGF1, E) WSL4.

E

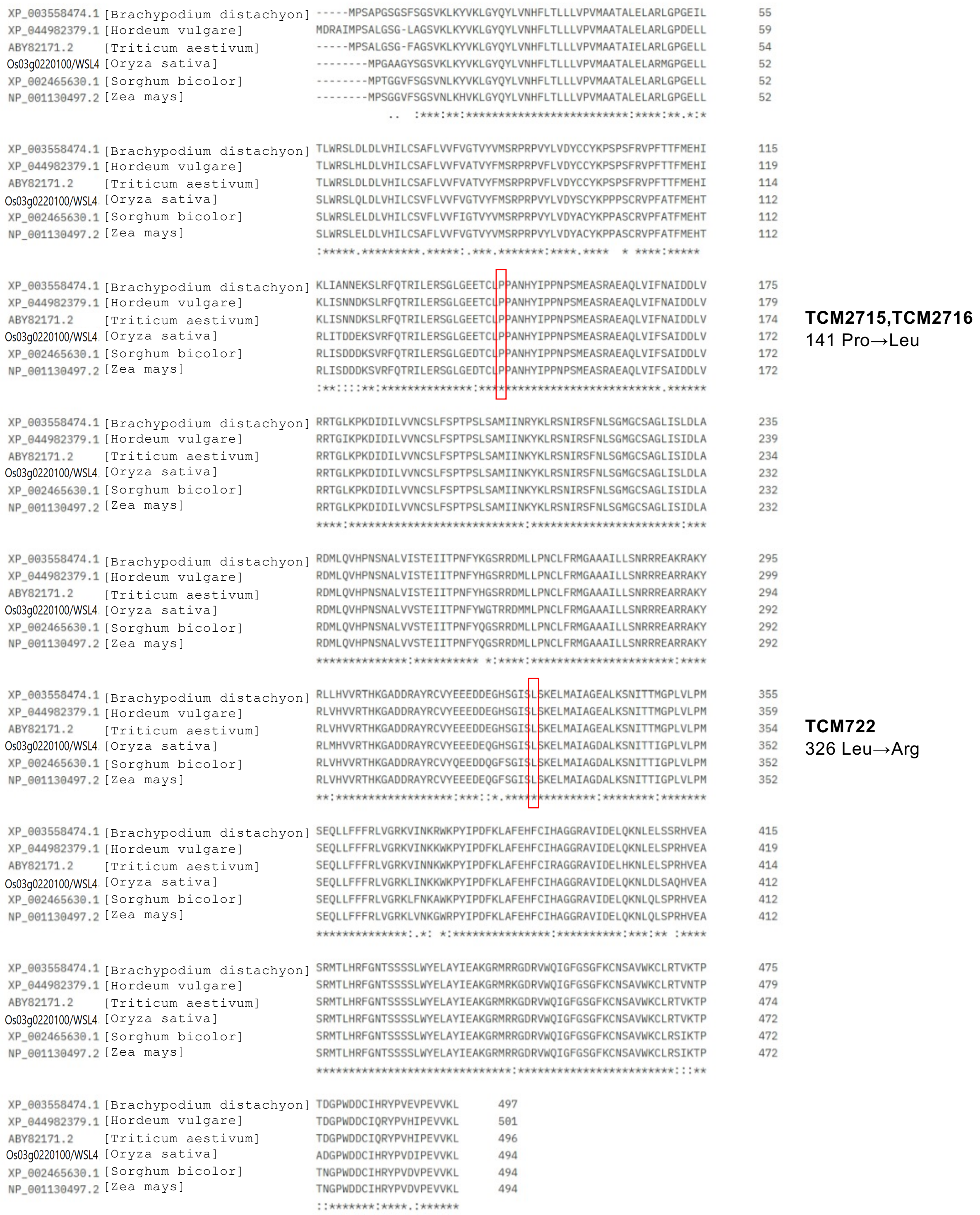

Supplementary Figure S3 (continued). Amino acid conservation of five proteins identified in this study as being involved in water repellency across the Poaceae. Amino acids highlighted with red boxes indicate the positions altered by gene mutations identified in this study. A) BGL, B) WSL3, C) WSL2, D) OsHSD1/LGF1, E) WSL4.

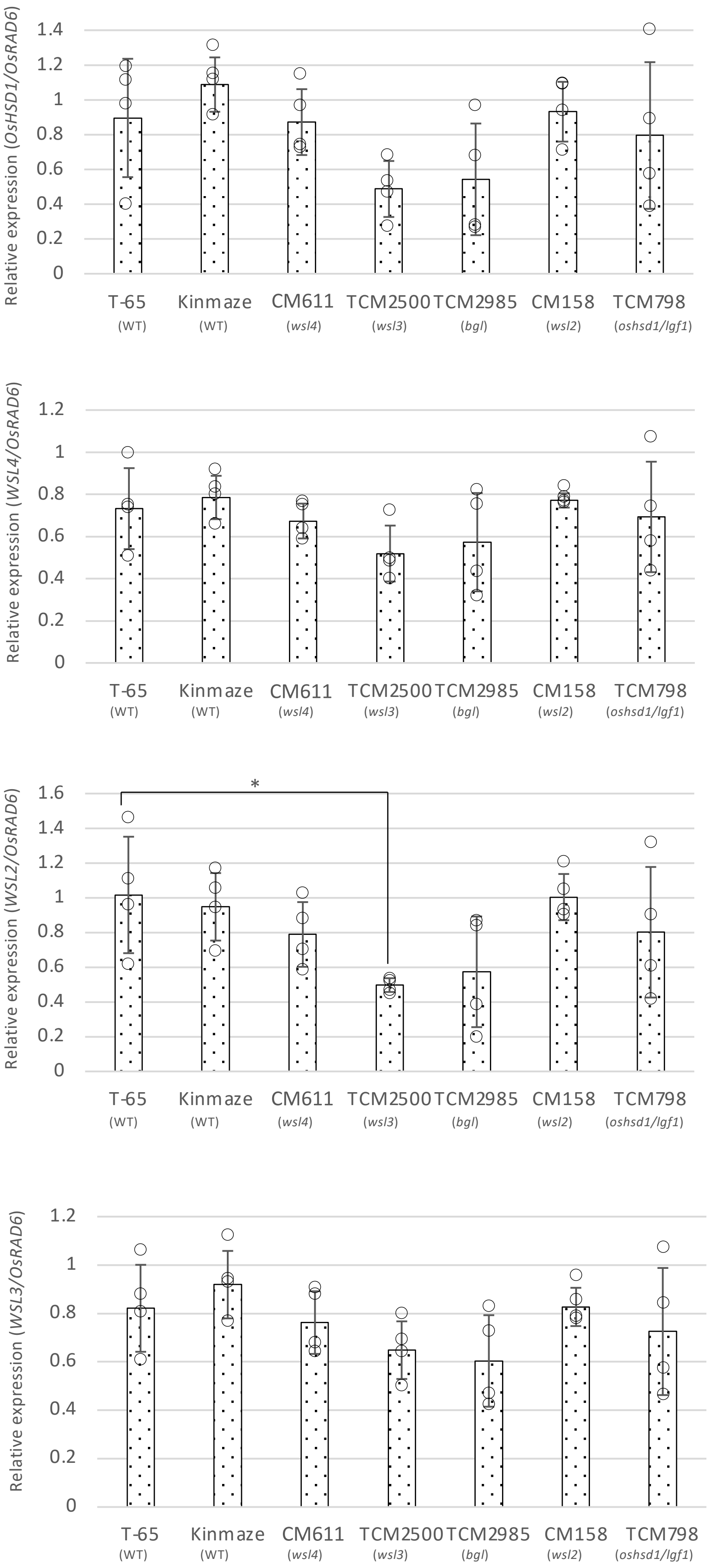

Supplementary Figure S4. Expression of wax synthesis genes in wetting leaf mutants. Relative expression level of *OsHSD1/LGF1*, *WSL4, WSL2, WSL3*. A ubiquitin-conjugating enzyme gene, *OsRAD6,* was used as an internal control. Single asterisks indicate a statistically significant difference compared to WT (t-test, $P < 0.05$).

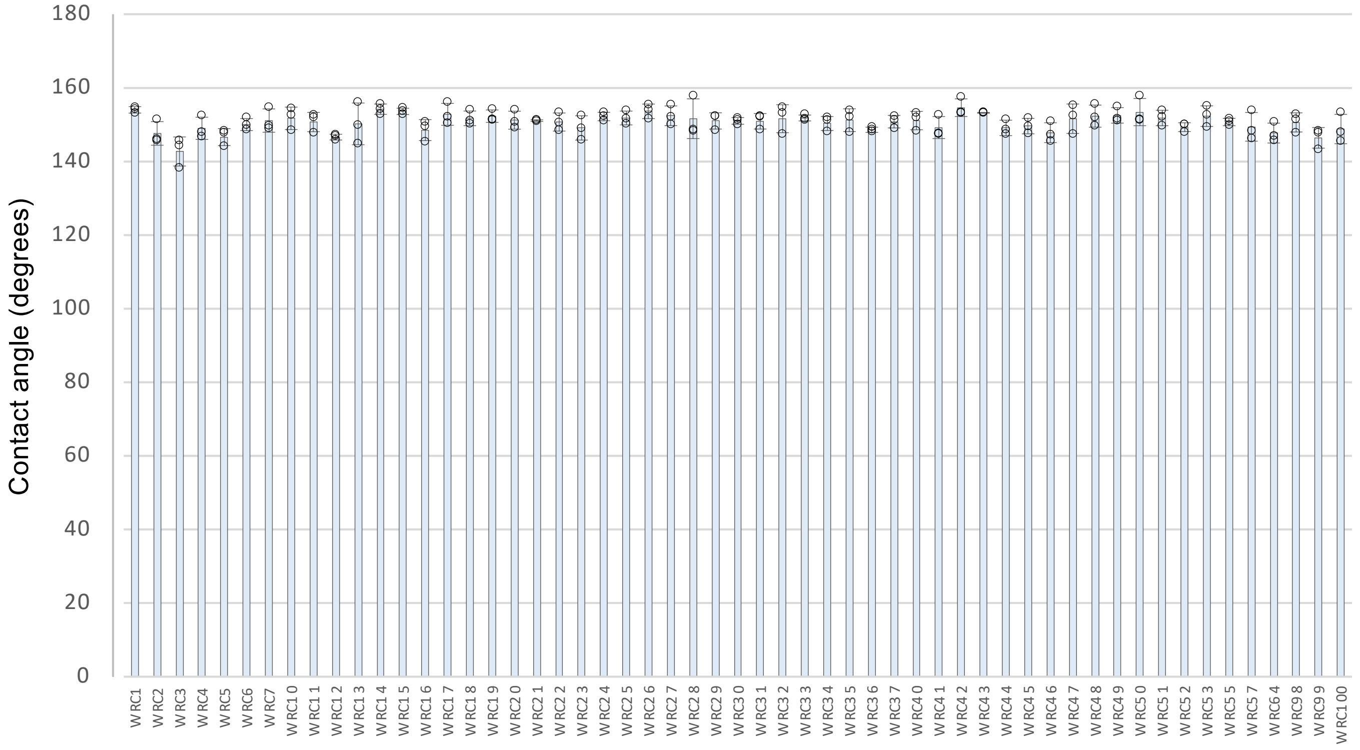

Supplementary Figure S5. Contact angles of the adaxial leaf blade of 4th leaf in 55 WRC accessions. Error bars indicate standard deviation (*n* = 3).

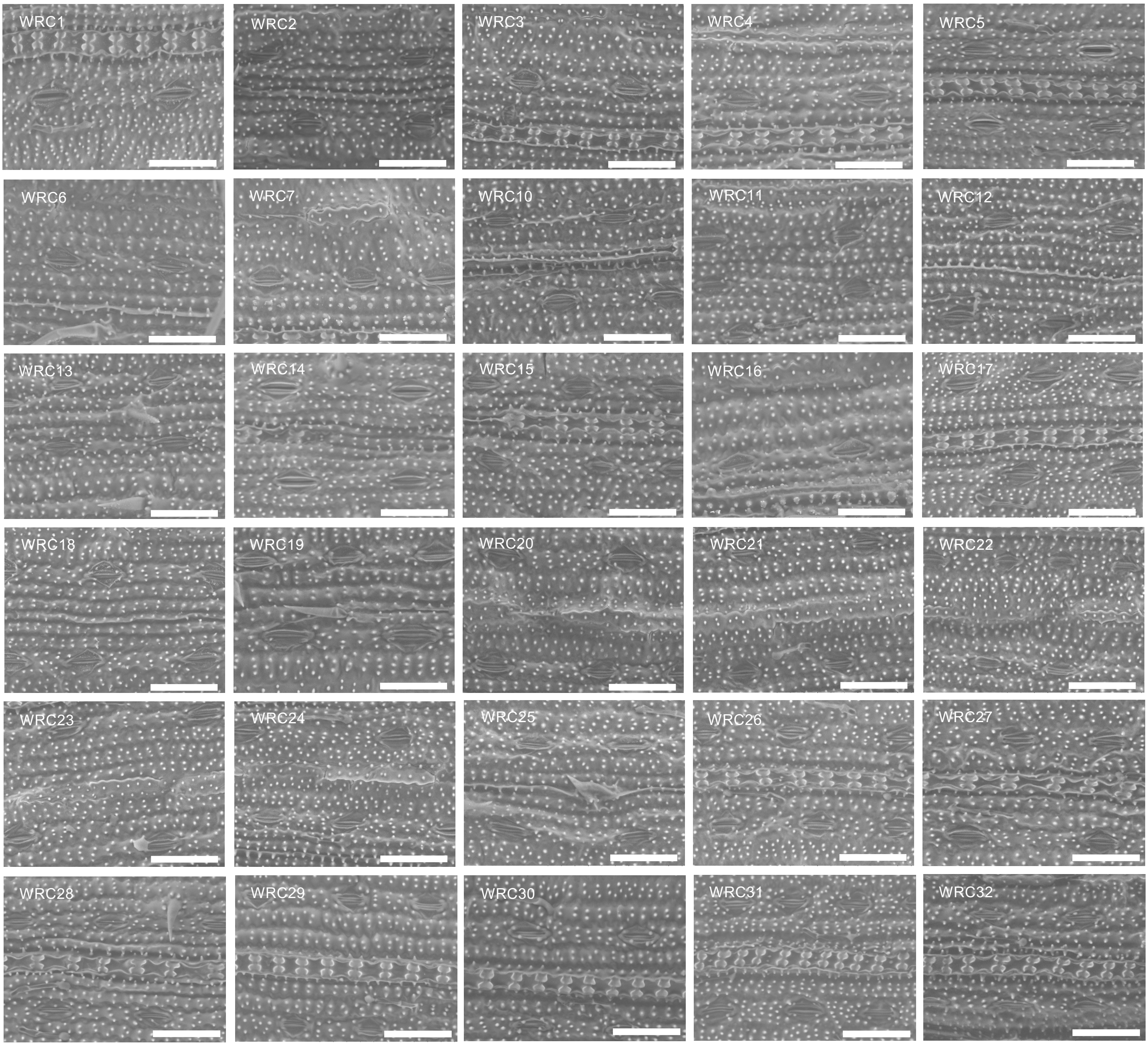

Supplementary Figure S6. Epidermal structure of WRC accessions by low-vacuum SEM. For each panel, the accession name is indicated in the top left. Scale bars = 50μm.

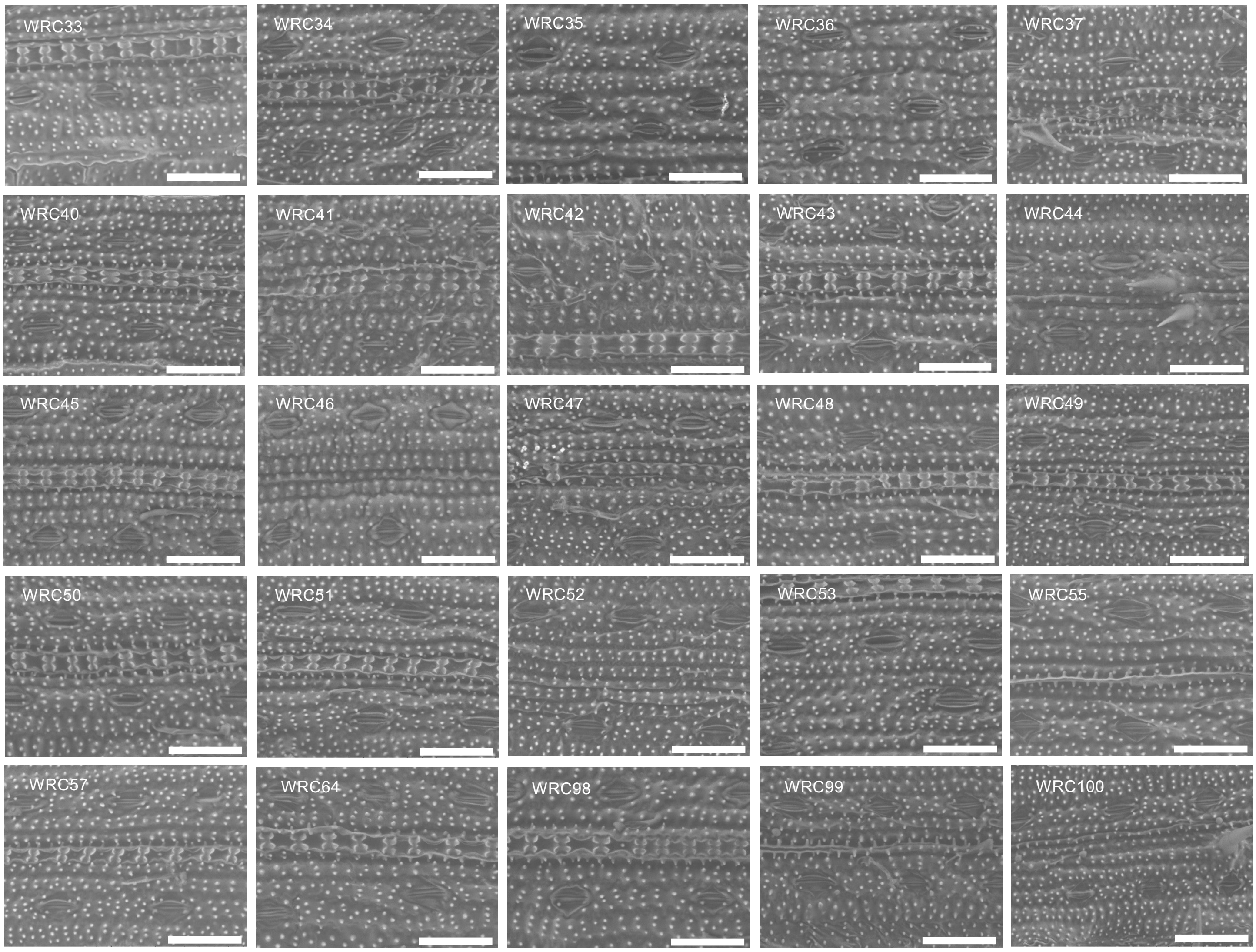

Supplementary Figure S6 (continued). Epidermal structure of WRC accessions by low-vacuum SEM. For each panel, the accession name is indicated in the top left. Scale bars = 50µm.

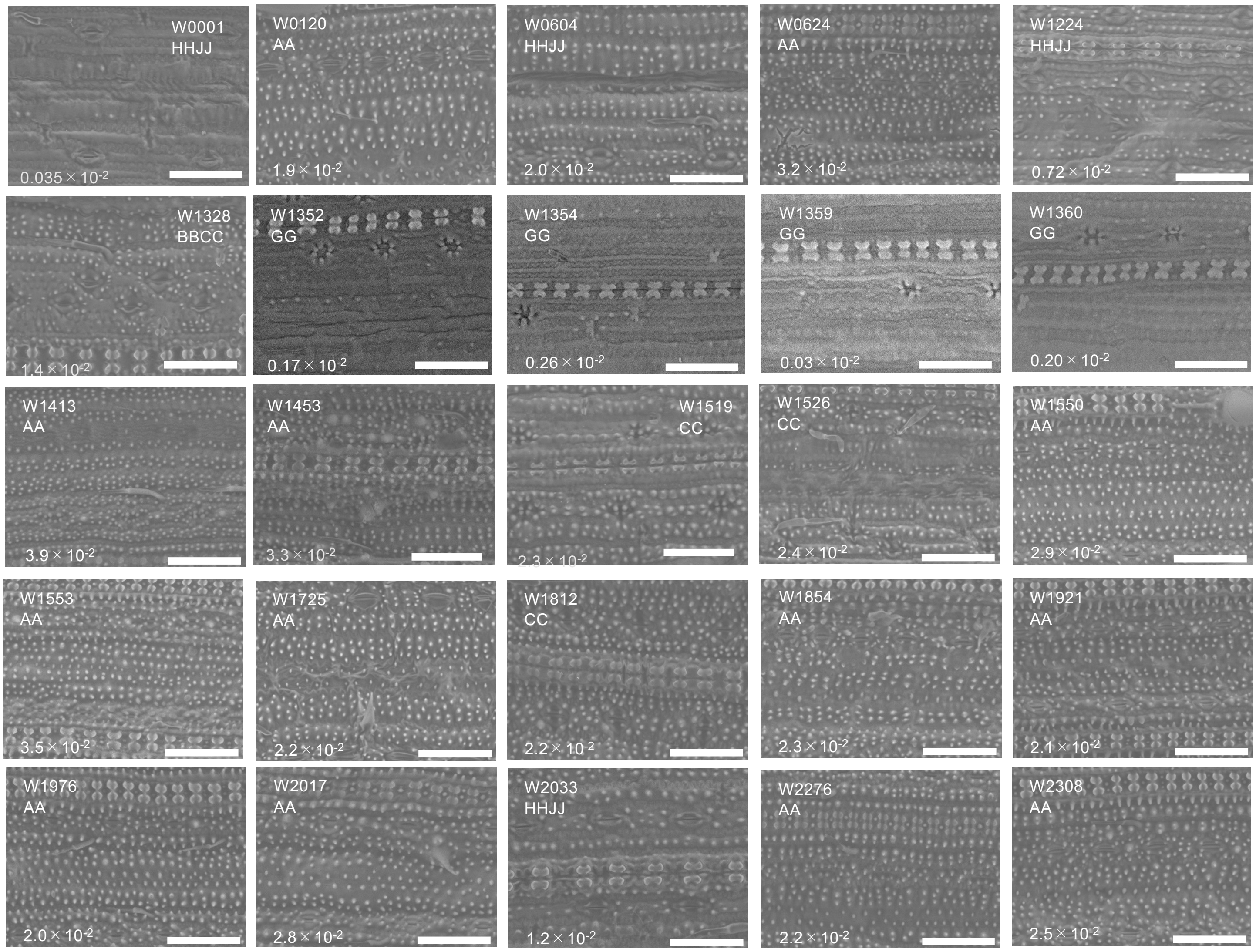

Supplementary Figure S7. Epidermal structure of wild *Oryza* accessions by low-vacuum SEM. For each panel, accession names and genome types are indicated in the top left corner and papillae density (papillae/μm^2) is shown in the bottom left corner. Scale bars = 50 µm. Scale bars = 50 µm.
